# EF1025, a Hypothetical Protein From *Enterococcus faecalis*, Interacts With DivIVA and Affects Cell Length and Cell Shape

**DOI:** 10.3389/fmicb.2020.00083

**Published:** 2020-02-12

**Authors:** Kusum Sharma, Taranum Sultana, Mingmin Liao, Tanya E. S. Dahms, Jo-Anne R. Dillon

**Affiliations:** ^1^Department of Biochemistry, Microbiology and Immunology, University of Saskatchewan, Saskatoon, SK, Canada; ^2^Vaccine and Infectious Disease Organization – International Vaccine Centre, University of Saskatchewan, Saskatoon, SK, Canada; ^3^Department of Chemistry and Biochemistry, University of Regina, Regina, SK, Canada

**Keywords:** Gram-positive bacteria, *Enterococcus faecalis*, cell division, DivIVA, protein–protein interaction, *Bacillus subtilis*

## Abstract

DivIVA plays multifaceted roles in Gram-positive organisms through its association with various cell division and non-cell division proteins. We report a novel DivIVA interacting protein in *Enterococcus faecalis*, named EF1025 (encoded by *EF1025*), which is conserved in Gram-positive bacteria. The interaction of EF1025 with DivIVA_Ef_ was confirmed by Bacterial Two-Hybrid, Glutathione S-Transferase pull-down, and co-immunoprecipitation assays. EF1025, which contains a DNA binding domain and two Cystathionine β-Synthase (CBS) domains, forms a decamer mediated by the two CBS domains. Viable cells were recovered after insertional inactivation or deletion of *EF1025* only through complementation of *EF1025 in trans*. These cells were longer than the average length of *E. faecalis* cells and had distorted shapes. Overexpression of *EF1025* also resulted in cell elongation. Immuno-staining revealed comparable localization patterns of EF1025 and DivIVA_Ef_ in the later stages of division in *E. faecalis* cells. In summary, EF1025 is a novel DivIVA interacting protein influencing cell length and morphology in *E. faecalis*.

## Introduction

A key protein in Gram-positive bacteria is DivIVA which is implicated in cell division and other functions (Cha and Stewart, 1997; Ben-Yehuda et al., 2003; Fadda et al., 2003; Pinho and Errington, 2004; Ramirez-Arcos, 2005; Briley et al., 2011; Halbedel and Lewis, 2019). DivIVA self-interacts, oligomerizes and associates with a functionally different array of proteins in different Gram-positive bacteria (Halbedel and Lewis, 2019). In *Bacillus subtilis* (Bs), DivIVA_Bs_ functions as a mid-cell determinant by attracting the MinC/MinD protein complex to the cell poles, thereby preventing cell division at the polar region (Cha and Stewart, 1997; Edwards and Errington, 1997; Marston and Errington, 1999; Edwards et al., 2000; Karoui and Errington, 2001; Harry and Lewis, 2003). DivIVA_Bs_ also associates with the DNA binding protein RacA, which acts as a bridge between the *oriC* region and the cell poles, anchoring the chromosome at the poles during sporulation (Ben-Yehuda et al., 2003). In addition, DivIVA_Bs_ interacts with Spo0J, participating in chromosome segregation during sporulation (Ben-Yehuda et al., 2003; Wu and Errington, 2003; Perry and Edwards, 2006); with ComN which is involved in competence development (dos Santos et al., 2012); and, with Maf, a regulator of cell shape and division (Butler et al., 1993). The interaction between Maf and DivIVA_Bs_ arrests cell division in competent cells (Briley et al., 2011). DivIVA of *Corynebacterium glutamicum* interacts with RodA and ParB (Donovan et al., 2012; Sieger et al., 2013), which binds the origin of replication with ParA, resulting in chromosomal segregation (Mierzejewska and Jagura-Burdzy, 2012). DivIVA is involved in apical growth and control of cell polarity in *Streptomyces coelicolor* (Flärdh, 2010), by interacting with ParB to co-ordinate chromosomal segregation (Donczew et al., 2016). DivIVA in *Streptococcus pneumoniae* interacts with several proteins implicated in divisome formation, including FtsZ, FtsA, ZapA, FtsK and FtsI, FtsB, FtsQ and FtsW (Fadda et al., 2007). These studies highlight the diverse functionality of DivIVA in Gram-positive organisms. There is no information regarding DivIVA-associating proteins in *Enterococcus faecalis* (Ef).

*Enterococcus faecalis*, an opportunistic, commensal, Gram-positive, ovococcal pathogen is recognized for its resistance to multiple antibiotics and for causing hospital-acquired infections (Murray, 1990; Cross and Jacobs, 1996; Hidron et al., 2008a, b; Sievert et al., 2013). Enterococcal infections are potentially fatal, causing neonatal and wound infections, endocarditis, meningitis, and urinary tract infections (Hidron et al., 2008a, b; Torelli et al., 2017). Due to its ability to form biofilms, catheter-related urinary tract infections with *E. faecalis* are difficult to treat (Mohamed and Huang, 2007). To formulate new therapeutic agents and targets for resisting antibiotic resistant *E. faecalis* infections, a greater understanding of enterococcal biology, physiology and genetics is required.

*Enterococcus faecali*s contains DivIVA (Ramirez-Arcos, 2005). This research describes a novel DivIVA-interacting protein, EF1025, which was annotated as a hypothetical protein in *E. faecalis* strain V583 (Paulsen et al., 2003). EF1025, which is conserved in most Gram-positive bacteria, contains a DNA binding domain at its N-terminus and two highly conserved Cystathionine β-Synthase (CBS) domains at the central and C-terminal regions. Bacterial Two-Hybrid (B2H), Glutathione S-Transferase (GST) pull-down, and Co-immunoprecipitation (Co-IP) assays were used to demonstrate interaction between EF1025 and DivIVA_Ef_. EF1025 self-interacts and forms a decamer. It was not possible to obtain viable cells after the deletion or insertional inactivation of *EF1025* without *in trans* expression of the gene. These rescued cells grew more slowly than wild type *E. faecalis*. Scanning electron microscopy (SEM) and atomic force microscopy (AFM) revealed cell elongation and aberrant cell shape in rescued cells. Cell elongation was also observed in SEM images when *EF1025* was overexpressed in *E. faecalis* cells. Using an *E. coli* model, overexpression of *EF1025* in *E. coli* PB103 resulted in filamentation. Immunofluorescence microscopy showed that EF1025 localized comparably to DivIVA_Ef_ localization during the later stages of cell division.

## Materials and Methods

### Strains, Plasmids and Growth Conditions

Strains and plasmids used in this study are listed in Supplementary Tables S1, S2. *E. coli* XL1-Blue or DH5α were used as hosts for cloning. *E. coli* C41 (DE3) was used to overexpress cloned proteins, *E. coli* PB103 (de Boer et al., 1988) for heterologous overexpression of *E. faecalis* proteins, and *E. coli* R721 (Di Lallo et al., 2001, 2003) was used for the bacterial-two hybrid evaluations. *E. coli* strains were grown at 37°C in Luria-Bertani (LB) medium (Difco, Detroit, MI, United States) and antibiotics were included in the following concentrations as required: ampicillin (Amp) 100 μg/mL, kanamycin (Kan) 50 μg/mL and erythromycin (Ery) 125 μg/mL. *E. faecalis* JH2-2 (Jacob and Hobbs, 1974), the parental strain, was used for the preparation of genomic DNA. *E. faecalis* was cultured at 37°C without aeration in Brain Heart Infusion (BHI) broth (Difco, Detroit, MI, United States) and supplemented with appropriate antibiotics if required (Ramirez-Arcos, 2005; Rigden et al., 2008). *Saccharomyces cerevisiae* SFY526, used in yeast two-hybrid (Y2H) assays (Clontech Laboratories, Inc., Mountain View, CA, United States), was grown at 30°C for 2–4 days on yeast extract-peptone-dextrose-adenine medium (YPDA) or on appropriate synthetic dropout media (Yeast Protocols Handbook, Clontech).

### Bioinformatic Analysis

DNA sequences interacting with DivIVA_Ef_, identified after screening Y2H libraries of *E. faecalis* JH2-2 (Supplementary Methods) were blasted against the *E. faecalis* V583 genome (Paulsen et al., 2003) using NCBI BLAST^1^. A putative open reading frame, named *EF1025* (GenBank accession number NC_004668), was identified from the *E. faecalis* V583 genome. The upstream sequence of *EF1025* (∼ 480 bp) was analyzed for promoter prediction^2^ and the deduced amino acid sequence of EF1025 was ascertained using ProtParam^3^. Homologs of EF1025 were identified using BLASTp^4^ against the non-redundant protein sequences database. EF1025 was also analyzed by PROSITE (Sigrist et al., 2010)^5^ to identify functional domains. Transmembrane motifs in EF1025 were predicted using TMbase^6^ and potential coiled-coil structures were predicted using COILS^7^.

### EF1025-DivIVA_Ef_ Interactions in the Bacterial Two-Hybrid Assays (B2H)

The B2H system of Di Lallo et al. (2001, 2003) was used to investigate interactions between DivIVA_Ef_ and EF1025 and its various domains. This particular assay involves a hybrid repressor which recognizes a chimeric operator. Potential interacting proteins are cloned at the two chimeric regions at the C-terminus of the hybrid repressor. The dimerization of the heterologous proteins permits reconstitution of the hybrid repressor which recognizes the chimeric operator and downregulates the activity of the downstream reporter gene, *lacZ* (Di Lallo et al., 2001). Modified B2H vectors pcI434-L and pcIp22-L, containing a linker with multiple endonuclease restriction sites were used in B2H assays (Supplementary Table S2A) (Zou et al., 2017). *EF1025*, *EF1025-c* (encoding AA80-209 of EF1025) and *divIVA*_Ef_ were PCR-amplified from the *E. faecalis* JH2-2 using primers EF1025-F/R, EF1025C-F/R and CBdivIVA-F/R, respectively (Supplementary Table S3A) and cloned into the modified B2H vectors, resulting in plasmids pdivIVA22, pdivIVA434, pEF1025434, p22CBS1CBS2 and p434CBS1CBS2, respectively (Supplementary Table S2A). These plasmids were transformed into *E. coli* R721 alone or in combination (Di Lallo et al., 2001, 2003; Greco-Stewart et al., 2007). Freshly transformed single colonies were grown overnight in 4 mL LB medium supplemented with Amp 50 μg/mL and Kan 30 μg/mL. Cells were diluted 1:100 using fresh LB medium containing the same antibiotics and were incubated for ∼1 h at 37°C, followed by the addition of 0.1 mM isopropyl β-D-1-thiogalactopyranoside (IPTG). Cells were further incubated to mid-log phase (OD600 ∼0.5) at 37°C, harvested, and tested for β-galactosidase activity, as previously described (Di Lallo et al., 2001). Each experiment was performed in triplicate and the average percentage β-galactosidase activity was calculated.

### GST Pull-Down Assays

To create a GST-DivIVA_Ef_ fusion, *divIVA*_Ef_ was PCR-amplified from genomic DNA from *E. faecalis* JH2-2 (see Supplementary Methods) using primers IVA-5/IVA-11 (Supplementary Table S3B) (Ramirez-Arcos, 2005). The amplicon was cloned into pGEX-2T, generating plasmid pGST-Div (Supplementary Table S2B). *EF1025* was PCR-amplified from *E. faecalis* JH2-2 DNA using primers EF1025F-F/R (Supplementary Table S3B) and cloned into pET30a(+), resulting in plasmid pETEF1025 (Supplementary Table S2B). The two CBS domains, i.e. CBS1 and CBS2, of EF1025 were PCR-amplified from *E. faecalis* JH2-2 DNA using primers EF1025-CF/R and cloned into pET30a(+), resulting in plasmid pETEF1025CBS12 (Supplementary Tables S2B, S3B).

GST-DivIVA_Ef_, 6 × His-EF1025, or 6 × His-EF1025CBS12 fusions were overexpressed in *E. coli* C41 (DE3) (Ramirez-Arcos, 2005). The GST-DivIVA_Ef_ fusion protein was purified using GST affinity beads (GST-Bind Kit, Novagen, United States). 6 × His-EF1025 or 6 × His-EF1025CBS12 were purified from 200 mL log-phase growth of *E. coli* C41 by sonication in 5 mL interaction buffer (IB, 20 mM Tris/HCl pH 7.5, 10% glycerol, 50 mM KCl, 0.5 mM EDTA, 1% Triton X100, 1 mM DTT). The cell lysate was centrifuged and the supernatant (50 μL) was incubated with 20 μL GST-DivIVA_Ef_ bound beads, pre-equilibrated with IB buffer, at 4°C for 2 h. Beads were washed with cold IB buffer 3× and the retained protein was eluted using a 40 μL 1 × SDS loading buffer and heating at 95°C for 10 min. Eluted protein was separated by SDS-PAGE, followed by Western blot analysis using anti-6 × His monoclonal antibody (Biorad, United States). The same protocol was used to study the DivIVA_Ef_ and EF1025-CBS12 interaction. Purified GST protein was used as a control and was produced in *E. coli* C41 (DE3) from plasmid pGEX2T.

### Production of Anti-EF1025 Polyclonal Antibody

6 × His-EF1025 was overexpressed in *E. coli* C41DE3 from plasmid pETEF1025 (Supplementary Table S2B) and was purified as described previously (Ramirez-Arcos, 2005). Female New Zealand White rabbits were injected with ∼30 μg/mL purified 6 × His-EF1025 in Freund’s adjuvant (Sigma; v/v = 1:1) at the Animal Core Facility of the Vaccine and Infectious Diseases Organization (University of Saskatchewan) with a booster dose on day 21 after the initial injection. Polyclonal IgG antibody was purified by affinity purification of antiserum using Protein-A sepharose beads (Pharmacia Bioscience; Ramirez-Arcos, 2005). Antibody specificity was tested by western blotting assay using an *E. faecalis* JH2-2 whole cell protein extract which was prepared by sonicating 50 mL of cell culture and resuspending the cells in 2.5 mL of Tris buffer (Supplementary Figure S1). Previously prepared anti-DivIVA_Ef_ (Ramirez-Arcos, 2005) was used as a positive control.

### Co-immunoprecipitation (Co-IP)

An overnight culture of *E. faecalis* JH2-2 was diluted 1:100 in BHI broth and incubated for 16–20 h at 37°C without aeration. Two hundred mL were centrifuged at 10,000 rpm for 10 min and the pellet was re-suspended in 5 mL Co-IP buffer (25 mM HEPES pH7.9, 100 mM NaCl, 5% glycerol, 0.5 mM EDTA, 0.1% Triton X100, 1 mM DTT and 0.5 mM PMSF). The suspension was sonicated, on ice, 3×, for 30 s each, with an interval of 20 s. The cell lysate was centrifuged under the same conditions (above) and the supernatant was collected for Co-IP assays.

Protein-A Sepharose beads (Pharmacia Inc., Canada) were cross-linked with 20 μg of either anti-DivIVA_Ef_ or anti-EF1025 polyclonal antibody in 200 μL PBS as follows: antibody was incubated with 50 μL Protein-A Sepharose beads at room temperature (RT) for 1 h. Beads were washed with PBS once and then washed twice with 0.2 M sodium borate (pH 9.0). Dimethylpimelimdate (Sigma) was added to the beads to a final concentration of 20 mM and incubated for 30 min at RT to allow cross-linking. The reaction was stopped by adding 0.2 M ethanolamine (final concentration 20 mM) pH8.0 (Sigma) and incubating at RT for 2 h. Beads were then washed with PBS and stored at 4°C for later use. Prior to Co-IP, 20 μL antibody-bound beads were incubated with 10 mg/mL BSA overnight at 4°C to block non-specific binding sites. Beads were then equilibrated with Co-IP buffer and subsequently incubated with 200 μL of *E. faecalis* JH2-2 cell extract for 2 h at 4°C. After removing the supernatant, beads were washed with Co-IP buffer 3 × for 10 min each. Proteins retained on the beads were eluted in 80 μL 1 × SDS loading buffer, separated on 12% SDS-PAGE, and transferred onto a nitrocellulose membrane for Western blot assay. Blots were probed with either anti-DivIVA_Ef_ or anti-EF1025 polyclonal antibody. Beads alone or beads cross-linked with anti-MinC_Ng_ polyclonal antibody (Ramirez-Arcos et al., 2001) were used as negative controls.

### EF1025 Self-Interaction

To determine whether EF1025 self-interacts, and to map the sites responsible for self-interaction, the predicted functional domains of EF1025 were cloned, in different combinations, into Y2H vectors as follows: EF1025CBS12 (AA80-204) carrying CBS1 and CBS2 domains, NCBS1-EF1025 (AA6-137) containing the N-terminus HTH domain and CBS1 domain, CBS2-EF1025 (AA144-204) containing the CBS2 domain, and N-EF1025 (AA6-50) containing the N-terminus HTH domain. *E. faecalis* JH2-2 DNA was used as a template for PCR amplification of these fragments. Primers for the amplification of various fragments are described in Supplementary Table S3C. These amplicons were cloned into the vectors pGAD424 and pGBT9 resulting in plasmids pGADEF1025CBS12, pGBDEF1025CBS12, pGADEF1025NCBS1, pGBDEF1025NCBS1, pGADEF1025C BS2, pGBDEF1025CBS2, pGADEF1025-N, and pGBDEF1025-N, respectively (Supplementary Table S2C). Each plasmid construct was co-transformed with a plasmid expressing full-length EF1025 (e.g. pGADEF1025 or pGBDEF1025) into *S. cerevisiae* SFY526. Transformation efficiencies were calculated by plating 50 μL of diluted transformants on separate plates followed by counting the number of colonies produced. Transformants were selected on complete synthetic medium lacking leucine and tryptophan (SD-leu-trp) (Clontech). Transformation efficiencies were calculated by plating 50 μL of diluted transformants on separate plates followed by counting the number of colonies produced. After 3–4 days of incubation at 30°C, using a colony lift assay (Clontech), cells were screened for blue color development in the presence of 5-Bromo-4-chloro-3-indolyl-β-D-galactopyranoside (X-Gal, Sigma-Aldrich; St. Louis, MS) to study the self-interaction ability of EF1025. Positive clones were further subcultured in SD-leu-trp broth and a spectrophotometric assay for β-galactosidase activity, using the substrate *o*-nitrophenyl-β-D-galactopyranoside was performed (Ramirez-Arcos, 2005).

SEC-MALS, the combination of Size Exclusion Chromatography with Multi-Angle Light Scattering analysis (Wyatt Technology, United States), was used to determine the oligomerization state of EF1025. Using His-bind resin (Novagen, Canada), 1mg of purified 6 × His-EF1025 was loaded onto a Superdex 200 column (Biorad) equilibrated with a buffer comprising 50 mM Tris base, 400 mM NaCl, pH 7.4. A single peak, corresponding to EF1025 eluted by SEC, was detected by the MALS detector to estimate molar mass.

### Overexpression of *EF1025* in *E. faecalis* JH2-2

To overexpress *EF1025* in *E. faecalis* JH2-2, *EF1025* was cloned into pMSP3545 (Supplementary Table S2). pMSP3545 was first modified by introducing an Amp-encoding gene that was PCR amplified from pcDNA3.1(+) using primer pairs AmpF/R (Supplementary Table S3D), into pMSP3545 creating pMSP3545A (Supplementary Table S2D). Linkers LinkA/B (Supplementary Table S3D), which contained restriction sites *Bam*HI and *Nco*I, were ligated to the Amp gene amplicon prior to ligation in pMSP3545. pMSP3545A was electroporated into electrocompetent *E. faecalis* JH2-2 cells using previously described methods (Ramirez-Arcos, 2005) and colonies were selected on BHI supplemented with Ery (125 μg/mL), creating *E. faecalis* MK0. *E. faecalis* JH2-2 and *E. faecalis* MK0 served as controls for all electroporation experiments. *EF1025* and 80 bp upstream, which included the predicted promoter sequence, was PCR amplified using primers EF1025npF/R, and the amplicon was digested with *Nco*I and *Xba*I, purified and subcloned into pMSP3545A, digested with the same enzymes, creating pMSPEF1025A (Supplementary Table S2D). pMSPEF1025A was transformed into *E. coli* DH5α and transformants were selected for Amp resistance. Clones were confirmed for the presence of EF1025 using restriction digestion and PCR amplification with primers EF1025npF/R. pMSPEF1025A was electroporated into electrocompetent *E. faecalis* JH2-2 cells creating *E. faecalis* MK23 (Supplementary Table S1) using previous methods (Ramirez-Arcos, 2005). To ascertain whether EF1025 was expressed from its native promoter in pMSPEF1025A, pMSPEF1025-flag was created by fusing a flag-tag encoding sequence which was PCR amplified from pcDNA3.1(+) using primers flagF/R (Supplementary Table S3D). The amplicon was ligated in pMSPEF1025A downstream of *EF1025* and electroporated into electrocompetent *E. faecalis* JH2-2 cells to create *E. faecalis* MK24 (Supplementary Table S1). EF1025 expression from pMSPEF1025-flag in *E. faecalis* MK24 was evaluated using an anti-flag monoclonal antibody (GenScript, United States) by Western blot analysis. Whole cell extracts of both *E. faecalis* JH2-2, *E. faecalis* MK23 and *E. faecalis* MK24 were prepared for these blots. In a separate Western blot, anti-EF1025 antibody was used to compare EF1025 expression levels in the same strains.

### Complementation *of EF1025* Deletions and Insertional Mutants in *E. faecalis JH2-2*

Clones of insertionally inactivated or deleted EF1025 in *E. faecalis* JH2-2 could not be recovered unless EF1025 was expressed *in trans*. Therefore, *E. faecalis* JH2-2 was co-transformed both with plasmids expressing EF1025 (i.e. either pMSPEF1025-pro or pMSPEF1025A) and plasmid constructs designed to insertionally inactive (i.e. p3ERMEF1025::Kan) or delete (i.e. p3ERMΔEF1025::Cat) EF1025.

To create p3ERMEF1025::Kan, first the N-terminal sequence of *EF1025* (AA1-55) was PCR-amplified from *E. faecalis* JH2-2 using primers CBSDPF/CBS55R-Hind (Supplementary Table S3D). The amplicon was digested and ligated to predigested pUC18, resulting in pUCEF1025-N (Supplementary Table S2D). Then, a kanamycin cassette (*Kan*^R^) was PCR-amplified from pTCV-lac (Supplementary Table S2D; Poyart and Trieu-Cuot, 1997) with primers KanF/R (Supplementary Table S3D), and the amplicon was inserted into pUCEF1025-N at its *Hin*dIII/*Sma*I sites, producing plasmid pUCEF1025-N-Kan (Supplementary Table S2D). The C-terminal sequence of EF1025 (AA56-209) was PCR-amplified from *E. faecalis* JH2-2 with primers CBS55F-*Sma*I/EF1025-R-*Bam*HI (Supplementary Table S3D) and the amplicon was inserted into pUCEF1025-N-Kan creating the plasmid pUCEF1025::Kan (Supplementary Table S2D). Finally, pUCEF1025::Kan was digested with *Eco*RI and *Bam*HI, yielding a fragment containing *EF1025-N, Kan^R^* and *EF1025-C.* This fragment was ligated into p3ERM-H, creating the suicide vector p3ERMEF1025::Kan (Supplementary Table S2D; Ramirez-Arcos, 2005). This plasmid was electroporated into *E. faecalis* JH2-2 (Ramirez-Arcos, 2005) with selection attempted using BHI agar containing Kan 500 μg/mL and incubation at 37°C for 2–3 days. Transformants were never obtained after multiple attempts, so p3ERMEF1025::Kan was co-electroporated with the shuttle plasmid pMSPEF1025-Pro that expresses wild type *EF1025 in trans* from its native promoter (Supplementary Table S2D) into *E. faecalis* JH2-2 to create *E. faecalis* MJ26 (Supplementary Table S3C; Ramirez-Arcos, 2005). Transformants were selected on BHI supplemented with Ery (125 μg/mL) and Kan (500 μg/mL). For each electroporation experiment, we used *E. faecalis* JH2-2 and MK0 as controls for growth on BHI supplemented with erythromycin. *E. faecalis* JH2-2 failed to grow in the presence of erythromycin while *E. faecalis* MK0 grew well. To confirm that transformants contained both an insertionally inactivated chromosomal *EF1025* as well as *EF1025* expressed *in trans* from pMSPEF1025-pro in *E. faecalis* MJ26, primers mutF/Kan-R, KanF/KanR, EF1025-Pro/KanR and KanF/CBSDPR were used to amplify chromosomal and plasmid fragments, followed by DNA sequencing of all amplified fragments for confirmation of the insertion (Supplementary Table S3D).

To ensure that phenotypes observed in *E. faecalis* MJ26 were not caused by polar effects of the insertional mutagenesis of EF1025 on the downstream gene EF1026, qPCR was performed to study the expression of both genes (Supplementary Methods).

A second strategy to inactivate *EF1025* in *E. faecalis* JH2-2 involved the non-polar deletion of chromosomal *EF1025* (LeDeaux et al., 1997) by introduction of the suicide plasmid pERMΔEF1025::Cat. Partial overlapping flanking primers ppdkF/R-*Bam*HI (Supplementary Table S3D) were used to amplify 500 bp upstream (includes the native promoter of *EF1025*) of the start codon of *EF1025* and 500 bp downstream of the stop codon of *EF1025* using primers 1026F/R-*Eco*RI (Supplementary Table S3D) of *E. faecalis* JH2-2 DNA. A chloramphenicol cassette was amplified from pLemo (NEB) using primers CatF/R (Supplementary Table S3D). The three fragments were combined by overlap PCR amplification (Hussain and Chong, 2016), creating a fragment that contained the chloramphenicol cassette flanked by the 500 bp upstream fragment and 500 bp downstream fragment. The resultant fragment was purified, digested and ligated into p3ERM-H, creating the suicide vector p3ERMΔEF1025::Cat (Supplementary Table S2D). As no transformants were recovered after electroporation of p3ERMΔEF1025::Cat into *E. faecalis* JH2-2, this plasmid along with pMSPEF1025A (Supplementary Table S2D) were co-electroporated into *E. faecalis* JH2-2 (Shepard and Gilmore, 1995) creating *E. faecalis* MK12. Transformants were selected on BHI agar plates containing Chl 5 μg/mL and Ery 125 μg/mL, incubated at 37°C for 2–3 days. The deletion of *EF1025* in *E. faecalis* MK12 was confirmed by PCR-amplification using primers ppdkF/EF26b-R, mutF/EF26b-R, ppdkF/EF1025npR, EF1025npF/1026R, CatF/1026R and CatF/R (Supplementary Tables S3C,D) followed by DNA sequencing of these amplified fragments (data not shown).

*Enterococcus faecalis* JH2-2 did not grow at this concentration of chloramphenicol. As a positive control, p3ERMΔEF1025::Cat was electroporated into *E. coli* DH5α and transformants were selected on LB agar plates containing Chl 33 μg/mL after incubation for 24 h at 37°C.

### Microscopy

A SU8010 Cold Field Emission Ultra-High-Resolution scanning electron microscope (WCVM, University of Saskatchewan, Saskatoon, SK, Canada) was used to image *E. faecalis* strains JH2-2, MK0, MK12, MJ26, MK23, MK24 (Supplementary Table S1). Strains were cultured in BHI medium with or without appropriate antibiotics, without agitation, at 37°C, either overnight (∼20 h) or to stationary phase. Cells were fixed on poly-L-lysine coverslips, dehydrated in ethanol, critical point dried, sputter coated with gold and imaged (Ramirez-Arcos et al., 2001). Length measurements were performed across the poles of the diplococcal bacteria and the percentage of elongated cells was calculated by measuring the lengths of 110–250 cells.

A Hitachi HT7700 High Contrast High-Resolution Digital Transmission Electron Microscope (WCVM, University of Saskatchewan, Saskatoon, Saskatchewan) was used to image *E. faecalis* strains JH2-2 and MJ26 prepared as previously described (Ramirez-Arcos, 2005).

### Immuno-Fluorescence Microscopy of *E. faecalis* JH2-2

To visualize DivIVA_Ef_ and EF1025 localization, *E. faecalis* JH2-2 cells in exponential phase were collected and fixed using a procedure modified from Harry and Lewis (2003). One mL of cell culture was harvested and the resuspended pellet was fixed with 1 ml fixation buffer (2.5% paraformaldehyde, 0.03% glutaraldehyde in 30 mM sodium phosphate buffer pH 7.5) for 30 min, at RT, then for 2 h at 4°C. Cells were washed 3× with 1 × PBS and resuspended in 200 μL GTE (50 mM glucose, 20 mM Tris-HCl pH 7.5, 10 mM EDTA) to which a freshly prepared lysozyme solution (2 mg/mL) was added. This volume was transferred to and fixed on poly-L-lysine coated coverslips. Cells attached to the coverslips were blocked with BSA-PBST (3% bovine serum albumin [wt/vol] and 0.2% Triton X-100 [vol/vol] in PBS) for 2 h at RT. Cells were then incubated with either anti-DivIVA_Ef_ (1:200) or anti-EF1025 (1:100) in BSA-PBST for 3 h at RT. After washing with PBST, cells were incubated with a fluorescence-labeled secondary antibody (1:500 dilutions in BSA-PBST, goat anti-rabbit Alexa Fluor 488, Invitrogen) for 45 min. Images were acquired using U-M655 and U-M665 filters and processed using *InVitro* 3 and ImagePro 6.0 software (Media Cybernetics). Each experiment was performed 4× using 2 independent cell cultures, and about 300 cells were counted for each immuno-staining. Cells were also stained with DAPI (Thermo Fisher, CA) and were mounted and observed under a 100X oil immersion objective using an Olympus BX61 microscope with standard filters. DAPI-stained cells were divided into five cell division stages. Stage 1 was defined as a single cell with a central condensed chromosome. Stage 2 cells contained a segregating chromosome as the cell started to divide. Stages 3 and 4 defined by the presence of two newly replicated cells with segregated chromosomes. As the cell completed one round of cell division, Stage 5 comprised of two daughter cells with condensed DNA in the center. *E. faecalis* MWMR16 cells, containing point mutations in the coiled coil region of DivIVA_Ef_, were used as negative control (Rigden et al., 2008).

## Results

### Identification and *in silico* Analysis of a Novel DivIVA_Ef_ Interacting Protein in *E. faecalis*

**FIGURE 1:**
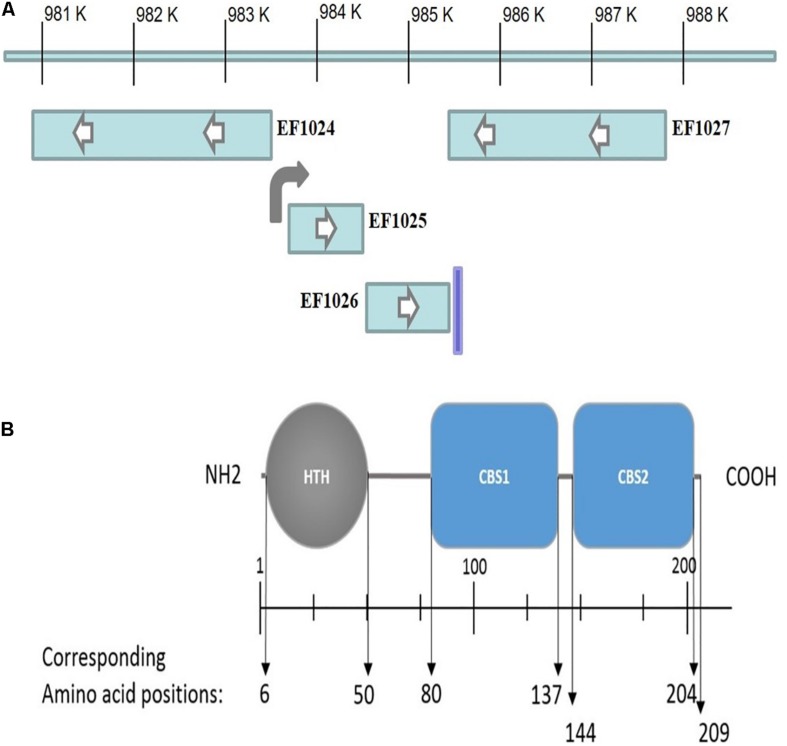
**(A)** *EF1025* position in *E. faecalis* V583 genome. Transcriptional orientation of genes upstream (i.e. EF1024) and downstream (i.e. EF1026 and EF1027) to *EF1025* (i.e. EF1025). The direction of an arrow within the rectangle indicates the transcriptional orientation of the gene. The bent arrow indicates promoter region upstream of EF1025 and vertical line indicates terminator region. **(B)** EF1025 domain prediction. N, N-terminus; C, C-terminus; HTH, Helix-turn-helix domain; CBS, Cystathionine-β-Synthase domain. Space in between domains constitutes hinge regions.

To identify DivIVA_Ef_ interacting proteins from *E. faecalis*, a Y2H system was used to screen an *E. faecalis* genomic DNA library using DivIVA_Ef_ as the bait protein (data not shown). Positive clones were sequenced and bioinformatic analysis indicated a sequence corresponding to the C-terminus of the hypothetical protein EF1025 (GenBank accession # NP_814759) of the *E. faecalis* V583 genome; *EF1025* spans nucleotide positions 983760-984389 (Figure 1A). *In silico* analysis of *EF1025* indicated that a ribosome binding site (GGAGG) is located at nucleotide position (nt) –6 to –10, and a putative promoter at position nt −36 to −87. *EF1025* has a transcriptional orientation (Figure 1B) similar to the downstream gene *EF1026*, a hypothetical protein with a kinase phosphoprotein phosphatase (PPPase) domain. A predicated terminator sequence is located downstream of *EF1026*. The upstream gene, *EF1024*, is transcribed in the opposite orientation of *EF1025* and *EF1026* and encodes a putative pyruvate phosphate dikinase (PPDK) domain (Figure 1B).

EF1025 comprises 209 amino acids (AA), with a molecular weight of ∼23 kDa and a theoretical isoelectric point of 6.75. Domain prediction studies (Figure 1B) showed that EF1025 contains an N-terminal Helix-turn-Helix (HTH) DNA binding domain (AA 6-50), and two CBS domains (i.e. CBS1, AA 80-137 and CBS2, AA 144-204). The CBS1 domain is in the central region of EF1025 and CBS2 is located at the C-terminus. EF1025 does not contain any transmembrane motifs (suggesting that it is a cytosolic protein), nor does it contain coiled-coil regions.

The EF1025 protein sequence was used as a query in BLASTp against 10000 targeted sequences in the non-redundant (nr) protein sequences database (last accessed May 2019). EF1025 was identified as belonging to the CBS pair superfamily and is conserved predominantly in Gram-positive bacteria, primarily in Firmicutes. As with EF1025, Gram-positive homologs contain an N-terminal HTH domain and two CBS domains located centrally and at the C-terminal. In *B*. *subtilis*, the EF1025 homolog is named CcpN and is involved in the gluconeogenic pathway (Servant et al., 2005).

### EF1025 Oligomerizes and Self-Interacts

**FIGURE 2:**
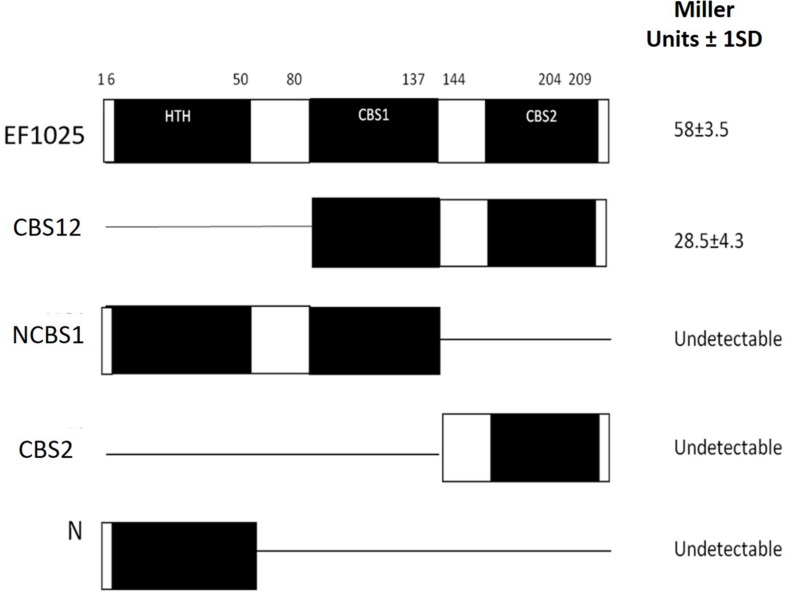
EF1025 self-interacts using its CBS1 and CBS2 domains. EF1025 self-interacts in the Y2H assay. Bars represent full-length and truncations of EF1025. Amino acid positions are indicated on the top. Open bars, predicted domains; closed bars, hinge regions; HTH, helix-turn-helix domain; CBS, cystathionine-β-synthase; Full-length EF1025 contains 209 amino acids (AA1-209); CBS12, EF1025 CBS1 and CBS2 domains together (AA80-204); NCBS1, N-terminus and CBS1 domain of EF1025 (AA1-131); CBS2, CBS2 domain of EF1025 (AA131-209); N, N-terminus of EF1025 (AA1-50). ND, not detectable. The experiment was conducted in triplicate. SD, standard deviation.

To determine whether EF1025 self-interacts, fragments comprising different combinations of domains of *EF1025* were cloned into Y2H vectors and initially tested for interactions using the colony lift assay (data not shown), followed by a quantitative assay for β-galactosidase activity. The quantitative assay indicated that EF1025 strongly self-interacts (Figure 2). Furthermore, the EF1025CBS12, containing the CBS1 and CBS2 domains, strongly interacted with EF1025. Fragments containing the N-terminus HTH domain and the central CBS1 domain (i.e. EF1025NCBS1) and fragments EF1025CBS2 (contains CBS2 domain) and EF1025-N (i.e. N-terminus HTH domain) showed no interaction with EF1025.

6×His-EF1025 was found to be a decamer, with an estimated molecular mass of 222 kDa, using a combination of Size Exclusion Chromatography (SEC) with Multi-Angle Light Scattering (MALS) analysis (Supplementary Figure S2). Reduced disulfide linkages, achieved by adding 1 mM Dithiothreitol, did not change the overall molecular weight of 6×His-EF1025.

### EF1025 Interacts With DivIVA_Ef_ *in vitro* and *in vivo*

**FIGURE 3:**
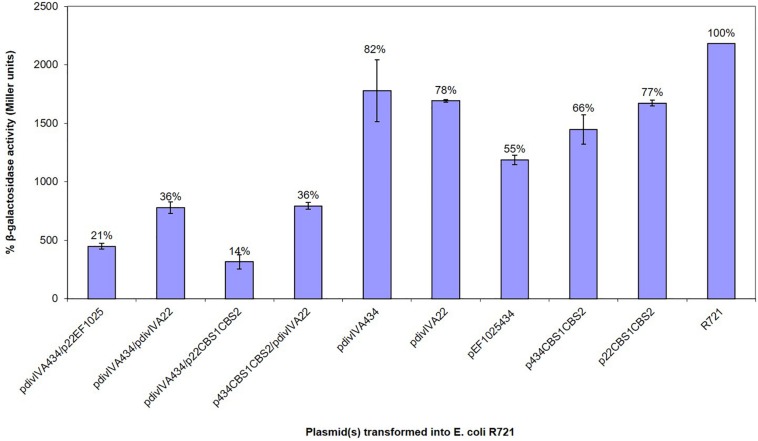
EF1025 interacts with DivIVA_Ef._ in B2H assay. The β-galactosidase activity was expressed in percentage Miller Units (*y*-axis). The *x*-axis shows the combination of B2H plasmids used in the experiment. Average values were obtained from three independent assays in triplicates. Values of less than 50% indicate a positive interaction. The error bars represent 1 standard deviation.

A B2H system was used to confirm preliminary Y2H results, showing the interaction of EF1025 with DivIVA_Ef_. In this assay, less than 50% residual β-galactosidase activity is indicative of a positive interaction (Di Lallo et al., 2001; Zou et al., 2017). *E. coli* R721 cells showed a baseline residual β-galactosidase activity of 100%. *E. coli* R721 transformed, with one of pdivIVA22, pdivIVA434, pEF1025434, p434CBS1CBS2, or p22CBS1CBS2, showed residual β-galactosidase activities of 78, 82, 55, 66, and 77%, respectively, and served as negative controls. The positive control (*E. coli* R721 cells containing plasmids pdivIVA22 and pdivIVA434), which demonstrated the self-interaction of DivIVA_Ef_ (Ramirez-Arcos, 2005), displayed 36% residual β-galactosidase activity. Our results indicate an interaction between DivIVA_Ef_ and EF1025 (Figure 3; pdivIVA434 and p22EF1025 together) with a residual β-galactosidase activity of 21%. The two CBS domains together (i.e. p22CBS1CBS2 or p434CBS1CBS2) also interacted with DivIVA_Ef_ (pdivIVA434 or pdivIVA22) with 14% residual β-galactosidase activity.

**FIGURE 4:**
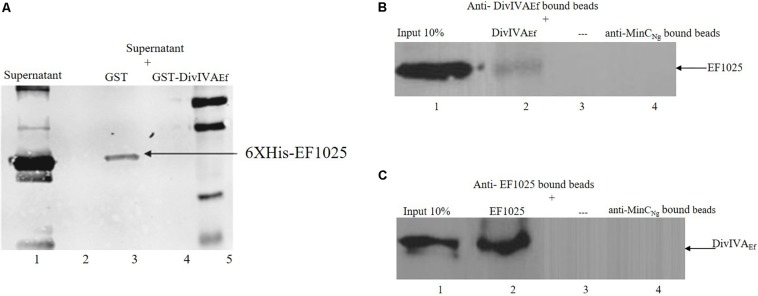
Interaction of EF1025 with DivIVA_Ef_. **(A)** GST pull-down assay. Shown is the Western blot probed with anti-6 × His (Bio-Rad, CA) monoclonal antibody to check the presence of EF1025. Lane 1: supernatant containing overexpressed EF1025 representing 10% input of EF1025; Lane 2: GST bound beads; Lane 3: GST-DivIVA_Ef_ bound beads; Lane 5: Protein ladder. **(B)** Co-immunoprecipitation assay of EF1025. EF1025 was co-precipitated with DivIVA_Ef_ using the anti-DivIVA_Ef_ antibody as bait. The blot was probed with the anti-EF1025 polyclonal antibody. Lane 1: *E. faecalis* extracts representing 10% input in Co-IP assays; Lane 2: anti-DivIVA_Ef_ antibody bound beads; Lane 3: beads alone; Lane 4: anti-MinC_Ng_ antibody bound beads. **(C)** Co-immunoprecipitation assay of DivIVA_Ef_. DivIVA_Ef_ with EF1025 using anti-EF1025 antibody as bait. The blot was probed with an anti-DivIVA_Ef_ polyclonal antibody. Lane 1: *E. faecalis* extracts representing 10% input in Co-IP assays; Lane 2: anti-EF1025 antibody bound beads; Lane 3: beads alone; Lane 4: anti-MinC_Ng_ antibody bound beads. — indicates the absence of any protein.

The interaction between EF1025 and DivIVA_Ef_ was also ascertained using a GST-pull down assay. A Western blot using anti-EF1025 antibody revealed that GST-DivIVA_Ef_ was pulled down by 6×His-EF1025 (Figure 4A, Lane 3) or 6×His-EF1025CBS12 (Supplementary Figure S3, Lane 3). GST did not interact with 6 × His-EF1025 (Figure 4A, Lane 2) or 6×His- EF1025-C (Supplementary Figure S3, Lane 2).

The *in vitro* interaction between EF1025 and DivIVA_Ef_ was also determined using a Co-IP assay. EF1025 co-precipitated with DivIVA_Ef_ using anti-DivIVA_Ef_ antibody (Figure 4B, Lane 2), and DivIVA_Ef_ co-precipitated with EF1025 with anti-EF1025 antibody (Figure 4C, Lane 2). As a negative control, anti-MinC_Ng_ (MinC from *N. gonorrhoeae*) antiserum failed to precipitate EF1025 or DivIVA_Ef_ (Figures 4B,C Lane 4).

### *In trans* Complementation of Inactivated or Deleted *EF1025*

Two strategies were used to inactivate or delete *EF1025* in *E. faecalis* JH2-2. First, we attempted to insert a *Kan*^R^ cassette at position nt151 (AA50 and Supplementary Figure S4A) of *EF1025* using p3ERMEF1025:Kan. No transformants were recovered after several attempts. The second strategy, in which an *EF1025* deletion mutant would be created by in frame replacement of *EF1025* (p3ERM EF1025:Cat) with a *Cat*^R^ cassette in *E. faecalis* JH2-2 also failed to produce transformant colonies. Expression of EF1025 was rescued by co-transformation with plasmid combinations p3ERMEF1025:Kan and pMSPEF1025-pro, and p3ERMΔEF1025:Cat and pMSPEF1025A. These rescue strategies were successful, creating transformant strains *E. faecalis* MJ26 and MK12, respectively (Supplementary Figure S4B). Taken together, the data suggest that *EF1025* may be an essential gene. *E. faecalis* MJ26 and MK12 grew more slowly than *E. faecalis* JH2-2 (Supplementary Figure S5).

The expression *EF1026* in *E. faecalis MJ26* was determined by RT-PCR in order to ascertain that the apparently lethal effects of the Kan^R^ insertion in *EF1025* was not due to polar effects on this gene. Amplified DNA fragments corresponding to the various regions of *EF1026* indicated that the gene was transcribed (Supplementary Figure S6). Expression levels (i.e. ΔC_T_ values) for *EF1026* in *E. faecalis* JH2-2 (i.e. 16.88 ± 0.13) and *E. faecalis* MJ26 (i.e. 16.79 ± 0.04) were equal.

**FIGURE 5:**
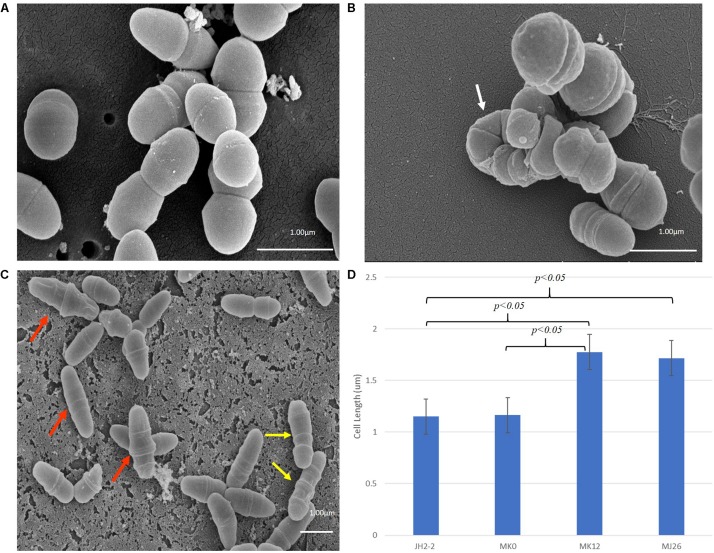
Rescued *E. faecalis* cells (i.e. *E. faecalis* MJ26 and MK12) showed compromised cell division phenotypes. Scanning electron micrographs showing **(A)** Normal *E. faecalis* JH2-2 lancet-shaped cells; **(B)** aggregated *E. faecalis* MJ26 cells with impaired segregation; **(C)** *E. faecalis* MK12 cells showing impaired cell shape and multiple division sites. White arrow indicates aggregated cells that failed to segregate; red arrows indicate cells with distorted cell shape; yellow arrows indicate cells with formation of multiple division rings. Bar scale indicated at the bottom right corner of each image; **(D)** Comparison of cell lengths for *E. faecalis* strains: JH2-2 (*n* = 141), MK0 (*n* = 165, harboring pMSPEA), MK12 (*n* = 127) and MJ26 (*n* = 174). *E. faecalis* strains JH2-2 and MK0 served as control strains. “n” represents the number of cells counted for each sample; two-tail *p* value from *t*-test for each group set has been indicated in the graph. All data was analyzed by *t*-test and ANOVA analysis, where *p*-value was < 0.05. The error bars represent 1 standard deviation.

**FIGURE 6:**
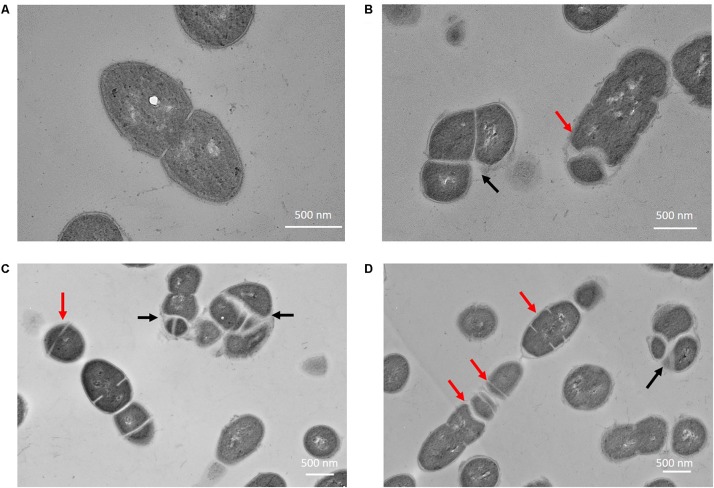
*Enterococcus faecalis* MJ26 cells showed impaired cell division. Transmission electron micrographs showing **(A)** wild-type *E. faecalis* JH2-2 lancet-shaped cells; **(B–D)** *E. faecalis* MJ26 cells with aggregated cells that failed to segregate and impaired septation leading to unequal daughter cells. Black arrows indicate aggregated cells that failed to segregate; red arrows indicate septa formation at random sites within the cells. Bar scale indicated at the bottom right corner of each image.

The phenotypes of *E. faecalis* MJ26 and MK12 differed from wild type *E. faecalis* JH2-2. SEM of *E. faecalis* JH2-2 showed cells with symmetrical division at the mid-cell with characteristic ovococcal cell morphology (Figures 5A, 6A). *E. faecalis* MJ26 and *E. faecalis* MK12 cells formed elongated cells with distorted cell shapes (Figures 5B,C) which were aggregated, failed to segregate (Figure 5B) and had multiple division sites within a single elongated cell (Figure 5C). Compared to the length of the wild type *E. faecalis* JH2-2 cells (1.16 ± 0.14 μm, *n* = 141), 47% of *E. faecalis* MJ26 (1.63 ± 0.29 μm, *n* = 174) and 49% of *E. faecalis* MK12 (1.74 ± 0.27 μm, *n* = 127) cells were significantly (*p* < 0.05) longer (Figure 5D) when measured across the poles. The control *E. faecalis* MK0 (i.e. contains empty plasmid pMSP3545A) had a cell length (1.15 ± 0.18 μm, *n* = 165) identical (*p* < 0.05) to *E. faecalis* JH2-2 (Figure 5D). Transmission electron microscopy showed that 10% of *E. faecalis* MJ26 cells were aggregated (*n* = 273) with abnormal septation, resulting in daughter cells of different sizes and shapes (Figures 6B–D). In addition, atomic force microscopy (AFM) (Supplementary Methods) showed larger aggregated cell clusters for *E. faecalis* MK12 as compared to *E. faecalis* JH2-2 (Supplementary Figure S7).

### Overexpression of *EF1025* in *E. faecalis* and *E. coli* Induces Cell Elongation

*Enterococcus faecalis* MK23 was created in which *EF1025* is expressed from its native promoter both from the chromosome and from pMSPEF1025A. In order to ensure that *EF1025* could be expressed from its native promoter *in trans*, *E. faecalis* MK24 was constructed (contains pMSPEF1025-flag) and the protein detected in whole cell extract by Western blot using a monoclonal anti-flag antibody (Supplementary Figure S8A. Lane 3). Expression of EF1025-flag was not detected in *E. faecalis* JH2-2 or MK23 cell extracts (Supplementary Figure S8A, Lanes 1 and 2). This confirmed expression of an extrachromosomal copy of EF1025 in *E. faecalis* MK24 when electroporated with pMSPEF1025-flag. This shows that *E. faecalis* MK23 also carries enhanced expression levels of EF1025 due to the presence of an extrachromosomal copy of EF1025. When anti-EF1025 antibody was used to identify the expression levels of EF1025, overexpression of EF1025 in *E. faecalis* MK23 and *E. faecalis* MK24 was observed as determined by densitometric quantification of band intensities, as compared to its expression in *E. faecalis* JH2-2 (Supplementary Figures S8B,C).

**FIGURE 7:**
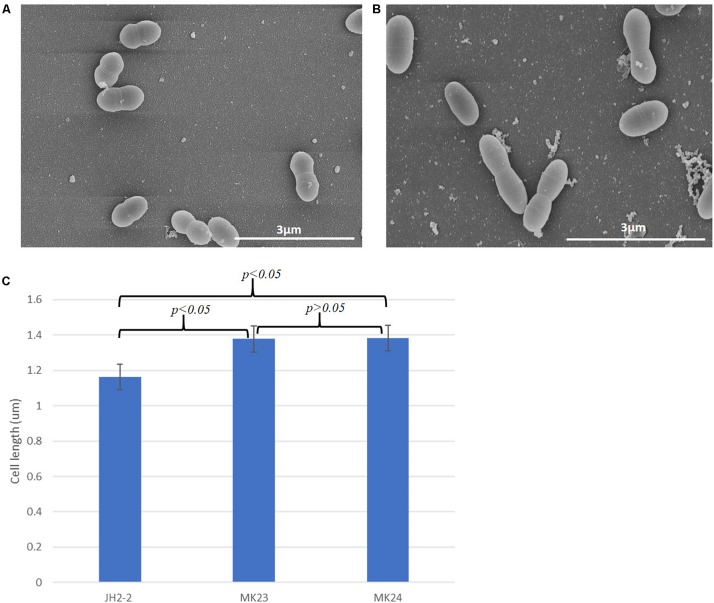
EF1025 overexpression in *E. faecalis* JH2-2 cells causes cell elongation. Scanning electron micrographs showing **(A)** *E. faecalis* JH2-2 lancet-shaped cells; **(B)** *E. faecalis* MK23 cells harboring pMSPEF1025A, showing elongated cell morphology. 3 μm bar scale at the bottom right corner of each image establishes the comparison in cell length for *E. faecalis* JH2-2 and MK23; and **(C)** Comparison of cell lengths of *E. faecalis* strains: JH2-2 (*n* = 141), MK23 (*n* = 202) and MK24 (*n* = 226) where “n” represents a number of cells counted for each sample; two-tail *p*-value from *t*-test for each group set has been indicated in the graph. All data was introduced to *t*-test and ANOVA analysis with a *p*-value < 0.05. The error bars represent 1 standard deviation.

SEM analysis showed a statistically significant (*p* < 0.05) increase in cell length (1.37 ± 0.21 μm, *n* = 202; Figures 7B,C) in *E. faecalis* MK23 as compared to wild type *E. faecalis* JH2-2 cells (1.16 ± 14 μm, *n* = 141; Figures 7A,C).

Seventy per cent of cells (63/89) overexpressing *EF1025* in *E. coli* PB103 (i.e. *E. coli* MK23) were filamentous (Supplementary Figure S9B) as compared to none of the control cells, i.e. *E. coli* cells with pUC18 and cells overexpressing *prgX_Ef_*, a transcriptional regulator encoding gene (Supplementary Figures S9A,C) (Christie and Dunny, 1986; Bae et al., 2000) in the same vector.

### EF1025 Localizes at the Septum and Cell Poles in *E. faecalis*

**FIGURE 8:**
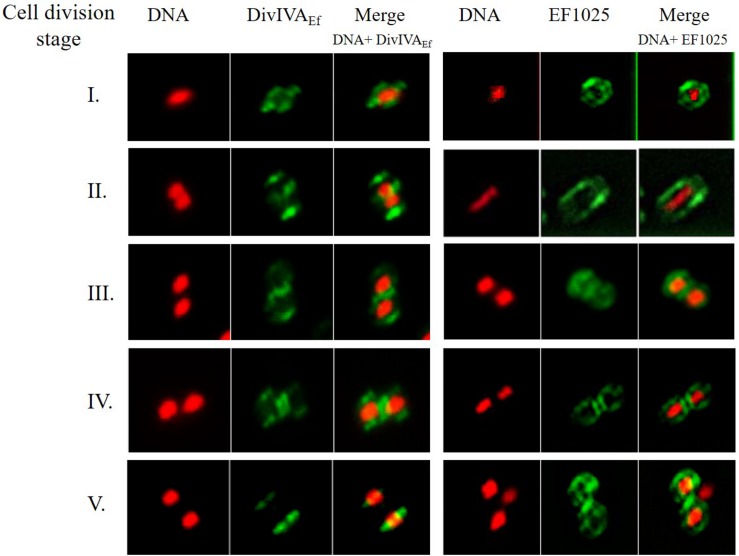
DivIVA_Ef_ and EF1025 localizes similarly in the later stages of cell division in *E. faecalis* JH2-2 cells. Averaged images and fluorescence intensity traces of *E. faecalis* JH2-2 cells grown to mid-exponential phase in BHI broth and dual-stained with DAPI and Alexa-Fluor 488 as described in the methodology section. Cells were segregated into five division Stages, and images from the indicated number of cells (n) were acquired using the *InVitro* 3 and ImagePro 6.0 softwares (Media Cybernetics) as described in Methodology. EF1025 localized at the cell poles and the septa in *E. faecalis* JH2-2 cells similar to DivIVA_Ef_ localization. Column 1 and 4, nucleoid localization from DAPI labeling; Column 2 and 5, DivIVA and EF1025 localization, respectively, in immunofluorescence microscopy; Column 3 and 6, merged image of DAPI stained nucleoid and fluorescent DivIVA_Ef_ and EF1025, respectively.

Immunofluorescence studies of *E. faecalis* JH2-2 cells, using anti-DivIVA_Ef_ or anti-EF1025 polyclonal antibody, determined the localization patterns of DivIVA_Ef_ and EF1025 during cell division. Cell division entailed 5 stages (273 cells counted for DivIVA_Ef_ and 281 for EF1025 localization). During Stage 1, as the cell started to divide and the chromosome started to segregate, DivIVAEf (20.5%, 56/273 cells) localized at the poles and along the length of the cell. In this stage, EF1025 (23.1%, 65/273 cells) was dispersed along the inner membrane (Figure 8, Stage 1). In Stage 2, EF1025 (14.9%, 42/281) localized along the length of the cell in contrast with DivIVA_Ef_ (36.7%, 100/273) that remained localized at the poles and the midcell (Figure 8, Stage 2). At Stage 3, EF1025 (36%, 104/281 cells) and DivIVA_Ef_ (16.1%, 44/273) localized similarly, i.e. to the cell poles and midcell. In Stage 4, as the cells progressed toward completion of cell division, EF1025 (13.2%, 37/281) and DivIVA_Ef_ (16.8%, 46/273) localized as disks and bands along the cell length and septum. With one completed round of cell division (i.e. Stage 5), EF1025 (11.7%, 33/281 cells) was redistributed along the inner membrane before another round of cell division, while DivIVA_Ef_ (9.9%, 27/73) once again localized as dots at the cell poles of the newly formed daughter cells (Figure 8, Stage 5), like Stage 1 cells. The coiled-coil region of DivIVA_Ef_ facilitates oligomerization and is essential for its biological functioning (Rigden et al., 2008). *E. faecalis* MWMR16 which contains point mutations in the coiled-coil region of DivIVA_Ef_ (Rigden et al., 2008) exhibited loss of DivIVA_Ef_ localization at the cell poles and midcell position (Supplementary Figure S10). The signal was observed to be dispersed all along the membrane and distinct stages of cell division were absent.

## Discussion

In the present study, we investigated a novel DivIVA_Ef_ interacting protein, EF1025, from *E. faecalis*. EF1025 belongs to the CBS pair superfamily and is conserved in Firmicutes including *Bacillus, Streptococcus, Clostridium, Paenibacillus, Staphylococcus, Lactobacillus, Streptomyces* and *Listeria*. Surprisingly, EF1025 homologs in the Firmicutes *S. pneumoniae, S. pyogenes and L. lactis* did not belong to the CBS pair superfamily as they contained an N-terminal HTH domain, but no CBS domains and their sequence similarities ranged from 40 to 44%. We also determined that EF1025 homologs, with uncharacterized functions and different combinations of CBS and HTH domains, may be present in species of the *Proteobacteria* and *Euryarcheota* such as *Vibrio, Campylobacter, Burkholderia, Acinetobacter, Fusobacterium, Methanosarcina*, and *Methanoculleus*. Proteins containing CBS domains are present in organisms ranging from archaea to humans and were originally identified in *Methanococcus jannaschii* as sequence motifs of approximately 60 amino acids (Bateman, 1997). Although several crystallographic studies have been carried out on CBS domains from bacteria, their precise function remains unexplained (Baykov et al., 2011). It has been postulated that CBS domains may act as allosteric “internal inhibitors” of the functional domains of proteins (Aravind and Koonin, 1999; Biemans-Oldehinkel et al., 2006). Proteins with CBS domains can form dimers through interaction of these domains. For example, TM0935 of *Thermotoga maritima* self-interacts through its two CBS domains forming a dimer (Miller et al., 2004). An Mg^2+^ transporter from *E. faecalis*, MgtE, also contains two CBS domains but the precise function of these domains remains unelucidated (Ragumani et al., 2010). Our experiments show the importance of the two CBS domains in EF1025 self-interaction. The absence of one CBS domain resulted in the loss of EF1025 self-interaction.

DivIVA, a topological factor in Gram-positive bacteria, interacts with a variety of proteins in various bacteria (Muchová et al., 2002; Halbedel and Lewis, 2019). The range of DivIVA interacting partners changes from one genus to another (Kaval and Halbedel, 2012). In *Listeria monocytogenes* (Lm), DivIVA_Lm_, performs a variety of functions through its interaction with different proteins (i.e. MinCD and SecA2), including precise positioning of the septum at midcell, assistance in the secretion of autolysins, and enabling swarming motility (Kaval et al., 2014, 2017). In *Streptococcus suis* (Ss) serotype 2, Ser/Thr kinases (STK) directly phosphorylate DivIVA_Ss_ thereby affecting cell growth and division (Nováková et al., 2010). DivIVA from *S. aureus* (Sa) associates with various divisome proteins (FtsZ_Sa_, FtsA_Sa_, EzrA_Sa_, DivIC_Sa_, DivIB_Sa_, PBP1_Sa_ and PBP2_Sa_) to ensure cell division and chromosome segregation (Bottomley et al., 2017). The molecular chaperone, DnaK, interacts and stabilizes DivIVA_Sa_ in *S. aureus* (Bukau and Walker, 1989; Bottomley et al., 2017). Bottomley et al. (2017) also reported an indirect function of DivIVA_Sa_ in chromosomal segregation by its interaction with the chromosome segregation protein, SMC (Bottomley et al., 2017). In *Mycobacterium smegmatis* (Ms) and *M. tuberculosis* (Mt), the DivIVA homolog is Wag31 (Nguyen et al., 2007; Kang et al., 2008; Meniche et al., 2014). Wag31_Mt_ interacts with the penicillin-binding protein, PBP3 (Mukherjee et al., 2009) as well as ParB (Donovan et al., 2012) and Wag31_Ms_ interacts with ParA (Donovan et al., 2012; Ginda et al., 2013). DivIVA from *E. faecalis* is essential for cell viability and growth, proper cell division and chromosome segregation (Ramirez-Arcos, 2005). Rigden et al. (2008) showed that the oligomerization of DivIVA_Ef_ is mediated by two centrally located coiled coils that are important for its proper biological functioning (Rigden et al., 2008). *E. faecalis* DivIVA_Ef_ mutant, *E. faecalis* MWMR16, contains a disrupted coiled coil region and failed to interact with EF1025 in a B2H assay due to the loss of a functional coiled-coil region in DivIVA_Ef_ (Rigden et al., 2008; Hedlin, 2009). Our research addressed the essentiality, localization and function of EF1025 during cell division.

Immunostaining showed that EF1025 localized in a pattern comparable to DivIVA_Ef_ in *E. faecalis*. Previously, Fadda et al. (2007) showed DivIVA localization to the mid-cell septa and poles in *S. pneumoniae* using similar methods (Fadda et al., 2007). EF1025 localized laterally along the cell length in Stages 1 and 2 and a pattern comparable to DivIVA_Ef_ in Stages 3, 4, and 5 of cell division. This localization progression may assist proper cell segregation required for cell division during the later stages of cell division when these two proteins interact. GpsB, an essential protein which determines the ellipsoidal shape in *S. pneumoniae*, localized in a similar but not identical manner to FtsZ, and is implicated in determining cell shape by septal ring closure (Land et al., 2013). There is a possibility that the localization of EF1025 (a cytosolic protein) to the lateral cell regions could be facilitated by DivIVA_Ef_ association. Different domains of DivIVA_Bs_ have been reported to interact with different partners that are membrane proteins as well as cytosolic proteins (Perry and Edwards, 2006; Bramkamp et al., 2008; Patrick and Kearns, 2008; Briley et al., 2011; dos Santos et al., 2012; Baarle et al., 2013; Halbedel et al., 2014; Schumacher, 2017; Halbedel and Lewis, 2019). Membrane localization of cytosolic proteins enhances the interaction abilities of interacting partners during processes such as cell division which involves multi-protein complex formation (Yogurtcu and Johnson, 2018).

We postulate that *EF1025* may be an essential gene since, during our attempts to delete or insertionally inactivate the gene, we were never able to recover viable cells. When these strains were complemented with EF1025 (i.e. *E. faecalis* MJ26 and MK12) they grew more slowly, with a longer log phase as compared to the *E. faecalis* JH2-2. The most likely explanation is that the rescue plasmids (i.e. pMSPEF1025-pro and pMSPEF1025A) failed to provide full complementation, which also led to altered cell shape and length. In *S. pneumoniae*, depletion of GpsB, caused cessation of growth and substantial cell elongation (Chastanet and Carballido-Lopez, 2012; Land et al., 2013). Based on the localization pattern of EF1025 and the elongated and aberrant phenotypes exhibited by *E. faecalis* MK12 cells, and the similarity of their localization patterns, we postulate that EF1025 could be one of the members of the septal machinery in *E. faecalis*, which has an unstudied GpsB homolog.

An interesting EF1025 homolog (41% identity) in *B. subtilis*, named CcpN (control catabolite protein of gluconeogenic genes), has two CBS domains and an HTH domain (Servant et al., 2005). CcpN plays a negative regulatory role in the transcription of the gluconeogenic genes *gapB* (one of the GAPDH-encoding genes) and *pckA* (encodes PEP carboxykinase), which are required in carbon catabolite repression pathways (Licht et al., 2005; Servant et al., 2005; Tännler et al., 2008; Licht and Brantl, 2009). Transcription regulation by CcpN has been attributed to its HTH domain which binds to the conserved upstream promoter regions of *gapB* and *pckA* (Licht et al., 2005; Servant et al., 2005; Tännler et al., 2008; Licht and Brantl, 2009). We detected strong interactions between CcpN and DivIVA_Bs_ by B2H and GST-pull down assay (paper in preparation). We observed that *gapB* from *B. subtilis* shared 48% homology with *type I gap* from *E. faecalis* while *pckA* from *B. subtilis* and *E. faecalis* showed 20% homology. *E. faecalis* was observed to have *type I* and *type II gap* as two homologs of *gapB*. Our preliminary sequence searches indicate that the conserved upstream promoter sequences from *B. subtilis* are absent in *E. faecalis* for *type I gapB* and *pckA* (unpublished data). This suggests that even though CcpN and EF1025 belong to the same superfamily, they possibly regulate the expression of different genes. CcpN is not an essential gene in contrast to EF1025 (Servant et al., 2005; Tännler et al., 2008), which may result from each protein regulating different genes.

In conclusion, this research presents the first evidence of a DivIVA_Ef_ interacting protein, EF1025, in *E. faecalis* that affects cell viability, cell length and shape. Using immunofluorescence, we showed that the localization patterns of EF1025 and DivIVA_Ef_ during the later stages of cell division in *E. faecalis* were similar. Our inability to insertionally inactivate or delete *EF1025* without in *trans* complementation of the gene indicates that gene is important for viability. Different microscopy methods showed cell elongation, aggregation and impaired cell division in complemented cells with a deleted or inactivated chromosomal gene.

## Data Availability Statement

The datasets generated for this study are available on request to the corresponding author.

## Author Contributions

KS designed and completed majority of the experiments, analyzed the results, and drafted the manuscript. ML performed and analyzed Y2H and other experiments. TS and TD designed and analyzed AFM experiments and edited the manuscript. J-AD directed the project and its implementation, analyzed the data, edited the manuscript drafts and approved the final submission in collaboration with all authors.

## Conflict of Interest

The authors declare that the research was conducted in the absence of any commercial or financial relationships that could be construed as a potential conflict of interest.

## Footnotes

1 http://blast.ncbi.nlm.nih.gov/Blast.cgi

2 http://www.fruitfly.org/seq_tools/promoter.html

3 http://us.expasy.org/tools/protparam.html

4 https://blast.ncbi.nlm.nih.gov/Blast.cgi?PAGE=Proteins

5 https://prosite.expasy.org/

6 http://www.ch.embnet.org/cgi-bin/TMPRED_form_parser

7 http://www.ch.embnet.org/software/COILS_form.html

## References

[B1] Aravind L. Koonin E. V. (1999). Gleaning non-trivial structural, functional and evolutionary information about proteins by iterative database searches11Edited by J. *M. Thornton. J. Mol. Biol.* 287 1023–1040. 10.1006/jmbi.1999.2653 10222208

[B2] Baarle S. van Celik I. N. Kaval K. G. Bramkamp M. Hamoen L. W. Halbedel S. (2013). Protein–protein interaction domains of *Bacillus subtilis* DivIVA. *J. Bacteriol.* 195 1012–1021. 10.1128/JB.02171-12 23264578 PMC3571322

[B3] Bae T. Clerc-Bardin S. Dunny G. M. (2000). Analysis of expression of *prgX*, a key negative regulator of the transfer of the *Enterococcus faecalis* pheromone-inducible plasmid pCF10. *J. Mol. Biol.* 297 861–875. 10.1006/jmbi.2000.3628 10736223

[B4] Bateman A. (1997). The structure of a domain common to archaebacteria and the homocystinuria disease protein. *Trends Biochem. Sci.* 22 12–13. 10.1016/s0968-0004(96)30046-7 9020585

[B5] Baykov A. A. Tuominen H. K. Lahti R. (2011). The CBS domain: a protein module with an emerging prominent role in regulation. *ACS Chem. Biol.* 6 1156–1163. 10.1021/cb200231c 21958115

[B6] Ben-Yehuda S. Rudner D. Z. Losick R. (2003). RacA, a bacterial protein that anchors chromosomes to the cell poles. *Science* 299 532–536. 10.1126/science.1079914 12493822

[B7] Biemans-Oldehinkel E. Mahmood N. A. B. N. Poolman B. (2006). A sensor for intracellular ionic strength. *Proc. Natl. Acad. Sci. U.S.A*, 103 10624–10629. 10.1073/pnas.0603871103 16815971 PMC1502282

[B8] Bottomley A. L. Liew A. T. F. Kusuma K. D. Peterson E. Seidel L. Foster S. J. (2017). Coordination of chromosome segregation and cell division in Staphylococcus aureus. *Front. Microbiol* . 8:1575. 10.3389/fmicb.2017.01575 28878745 PMC5572376

[B9] Bramkamp M. Emmins R. Weston L. Donovan C. Daniel R. A. Errington J. (2008). A novel component of the division-site selection system of *Bacillus subtilis* and a new mode of action for the division inhibitor MinCD. *Mol. Microbiol.* 70 1556–1569. 10.1111/j.1365-2958.2008.06501.x 19019154

[B10] Briley K. Prepiak P. Dias M. J. Hahn J. Dubnau D. (2011). Maf acts downstream of ComGA to arrest cell division in competent cells of *B. subtilis*. *Mol. Microbiol.* 81 23–39. 10.1111/j.1365-2958.2011.07695.x 21564336 PMC3781949

[B11] Bukau B. Walker G. C. (1989). Delta dnaK52 mutants of *Escherichia coli* have defects in chromosome segregation and plasmid maintenance at normal growth temperatures. *J. Bacteriol.* 171 6030–6038. 10.1128/jb.171.11.6030-6038.1989 2681151 PMC210468

[B12] Butler Y. X. Abhayawardhane Y. Stewart G. C. (1993). Amplification of the *Bacillus subtilis* maf gene results in arrested septum formation. *J. Bacteriol.* 175 3139–3145. 10.1128/jb.175.10.3139-3145.1993 8387996 PMC204636

[B13] Cha J.-H. Stewart G. C. (1997). The divIVA minicell locus of *Bacillus subtilis*. *J. Bacteriol.* 179 1671–1683. 10.1128/jb.179.5.1671-1683.1997 9045828 PMC178881

[B14] Chastanet A. Carballido-Lopez R. (2012). The actin-like MreB proteins in *Bacillus subtilis*: a new turn. *Front. Biosci. Sch. Ed.* 4:1582–1606. 10.2741/s354 22652894

[B15] Christie P. J. Dunny G. M. (1986). Identification of regions of the *Streptococcus faecalis* plasmid pCF-10 that encode antibiotic resistance and pheromone response functions. *Plasmid* 15 230–241. 10.1016/0147-619X(86)90041-7 3012615

[B16] Cross J. T. Jacobs R. F. (1996). Vancomycin-resistant enterococcal infections. *Semin. Pediatr. Infect. Dis.* 7 162–169. 10.1016/S1045-1870(96)80004-5

[B17] de Boer P. A. Crossley R. E. Rothfield L. I. (1988). Isolation and properties of minB, a complex genetic locus involved in correct placement of the division site in *Escherichia coli*. *J. Bacteriol.* 170 2106–2112. 10.1128/jb.170.5.2106-2112.1988 2834323 PMC211093

[B18] Di Lallo G. Castagnoli L. Ghelardini P. Paolozzi L. (2001). A two-hybrid system based on chimeric operator recognition for studying protein homo/heterodimerization in *Escherichia coli*. *Microbiol. Read. Engl.* 147 1651–1656. 10.1099/00221287-147-6-1651 11390696

[B19] Di Lallo G. Fagioli M. Barionovi D. Ghelardini P. Paolozzi L. (2003). Use of a two-hybrid assay to study the assembly of a complex multicomponent protein machinery: bacterial septosome differentiation. *Microbiol. Read. Engl.* 149 3353–3359. 10.1099/mic.0.26580-0 14663069

[B20] Donczew M. Mackiewicz P. Wróbel A. Flärdh K. Zakrzewska-Czerwińska J. Jakimowicz D. (2016). ParA and ParB coordinate chromosome segregation with cell elongation and division during *Streptomyces sporulation*. *Open Biol* 6:150263. 10.1098/rsob.150263 27248800 PMC4852455

[B21] Donovan C. Sieger B. Krämer R. Bramkamp M. (2012). A synthetic *Escherichia coli* system identifies a conserved origin tethering factor in Actinobacteria. *Mol. Microbiol.* 84 105–116. 10.1111/j.1365-2958.2012.08011.x 22340668

[B22] dos Santos V. T. Bisson-Filho A. W. Gueiros-Filho F. J. (2012). DivIVA-mediated polar localization of ComN, a posttranscriptional regulator of *Bacillus subtilis*. *J. Bacteriol.* 194 3661–3669. 10.1128/JB.05879-11 22582279 PMC3393515

[B23] Edwards D. H. Errington J. (1997). The *Bacillus subtilis* DivIVA protein targets to the division septum and controls the site specificity of cell division. *Mol. Microbiol.* 24 905–915. 10.1046/j.1365-2958.1997.3811764.x 9219999

[B24] Edwards D. H. Thomaides H. B. Errington J. (2000). Promiscuous targeting of *Bacillus subtilis* cell division protein DivIVA to division sites in *Escherichia coli* and fission yeast. *EMBO J.* 19 2719–2727. 10.1093/emboj/19.11.2719 10835369 PMC212753

[B25] Fadda D. Pischedda C. Caldara F. Whalen M. B. Anderluzzi D. Domenici E. (2003). Characterization of divIVA and other genes located in the chromosomal region downstream of the dcw cluster in *Streptococcus pneumoniae*. *J. Bacteriol.* 185 6209–6214. 10.1128/JB.185.20.6209-6214.2003 14526035 PMC225046

[B26] Fadda D. Santona A. D’Ulisse V. Ghelardini P. Ennas M. G. Whalen M. B. (2007). *Streptococcus pneumoniae* DivIVA: localization and interactions in a MinCD-free context. *J. Bacteriol.* 189 1288–1298. 10.1128/JB.01168-06 17098892 PMC1797354

[B27] Flärdh K. (2010). Cell polarity and the control of apical growth in Streptomyces. *Curr. Opin. Microbiol.* 13 758–765. 10.1016/j.mib.2010.10.002 21036658

[B28] Ginda K. Bezulska M. Ziółkiewicz M. Dziadek J. Zakrzewska-Czerwińska J. Jakimowicz D. (2013). ParA of *Mycobacterium smegmatis* co-ordinates chromosome segregation with the cell cycle and interacts with the polar growth determinant DivIVA. *Mol. Microbiol.* 87 998–1012. 10.1111/mmi.12146 23289458

[B29] Greco-Stewart V. Ramirez-Arcos S. Liao M. Dillon J. R. (2007). N terminus determinants of MinC from *Neisseria gonorrhoeae* mediate interaction with FtsZ but do not affect interaction with MinD or homodimerization. *Arch. Microbiol.* 187 451–458. 10.1007/s00203-007-0210-4 17287984

[B30] Halbedel S. Kawai M. Breitling R. Hamoen L. W. (2014). SecA is required for membrane targeting of the cell division protein DivIVA in vivo. *Front. Microbiol* 5:58. 10.3389/fmicb.2014.00058 24592260 PMC3924036

[B31] Halbedel S. Lewis R. J. (2019). Structural basis for interaction of DivIVA/GpsB proteins with their ligands. *Mol. Microbiol.* 111 1404–1415. 10.1111/mmi.14244 30887576

[B32] Harry E. J. Lewis P. J. (2003). Early targeting of Min proteins to the cell poles in germinated spores of *Bacillus subtilis*: evidence for division apparatus-independent recruitment of Min proteins to the division site. *Mol. Microbiol.* 47 37–48. 10.1046/j.1365-2958.2003.03253.x 12492852

[B33] Hedlin C. E. (2009). *The Essentiality of DivIVAEf Oligomerization for Proper Cell Division in Enterococcus faecalis and Interaction With a Novel Cell Division Protein.* Available at: https://harvest.usask.ca/handle/10388/etd-04152009-115838 (accessed November 8, 2019).

[B34] Hidron A. I. Edwards J. R. Patel J. Horan T. C. Sievert D. M. Pollock D. A. (2008a). Antimicrobial-resistant pathogens associated with healthcare-associated infections: annual summary of data reported to the national healthcare safety network at the centers for disease control and prevention, 2006–2007. *Infect. Control Hosp. Epidemiol.* 29 996–1011. 10.1086/591861 18947320

[B35] Hidron A. I. Schuetz A. N. Nolte F. S. Gould C. V. Osborn M. K. (2008b). Daptomycin resistance in *Enterococcus faecalis* prosthetic valve endocarditis. *J. Antimicrob. Chemother.* 61 1394–1396. 10.1093/jac/dkn105 18344547

[B36] Hussain H. Chong N. F.-M. (2016). Combined overlap extension PCR Method for improved site directed mutagenesis. *BioMed Res. Int* 2016 1–7. 10.1155/2016/8041532 27995143 PMC5138438

[B37] Jacob A. E. Hobbs S. J. (1974). Conjugal transfer of plasmid-borne multiple antibiotic resistance in *Streptococcus faecalis* var. zymogenes. *J. Bacteriol.* 117 360–372. 10.1128/jb.117.2.360-372.1974 4204433 PMC285522

[B38] Kang C.-M. Nyayapathy S. Lee J.-Y. Suh J.-W. Husson R. N. (2008). Wag31, a homologue of the cell division protein DivIVA, regulates growth, morphology and polar cell wall synthesis in mycobacteria. *Microbiology* 154 725–735. 10.1099/mic.0.2007/014076-0 18310019

[B39] Karoui M. E. Errington J. (2001). Isolation and characterization of topological specificity mutants of minD in *Bacillus subtilis*. *Mol. Microbiol.* 42 1211–1221. 10.1046/j.1365-2958.2001.02710.x 11886553

[B40] Kaval K. G. Halbedel S. (2012). Architecturally the same, but playing a different game. *Virulence* 3 406–407. 10.4161/viru.20747 22722244 PMC3478244

[B41] Kaval K. G. Rismondo J. Halbedel S. (2014). A function of DivIVA in *Listeria monocytogenes* division site selection. *Mol. Microbiol.* 94 637–654. 10.1111/mmi.12784 25185533

[B42] Kaval K. G. Hauf S. Rismondo J. Hahn B. Halbedel S. (2017). Genetic dissection of DivIVA functions in *Listeria monocytogenes*. *J. Bacteriol.* 199:e00421-17. 10.1128/JB.00421-17 28972021 PMC5686608

[B43] Land A. D. Tsui H.-C. T. Kocaoglu O. Vella S. A. Shaw S. L. Keen S. K. (2013). Requirement of essential Pbp2x and GpsB for septal ring closure in *Streptococcus pneumoniae* D39. *Mol. Microbiol.* 90 939–955. 10.1111/mmi.12408 24118410 PMC4120849

[B44] LeDeaux J. R. Solomon J. M. Grossman A. D. (1997). Analysis of non-polar deletion mutations in the genes of the spo0K (opp) operon of *Bacillus subtilis*. *FEMS Microbiol. Lett.* 153 63–69. 10.1111/j.1574-6968.1997.tb10464.x 9252573

[B45] Licht A. Brantl S. (2009). The transcriptional repressor CcpN from *Bacillus subtilis* uses different repression mechanisms at different promoters. *J. Biol. Chem.* 284 30032–30038. 10.1074/jbc.M109.033076 19726675 PMC2781557

[B46] Licht A. Preis S. Brantl S. (2005). Implication of CcpN in the regulation of a novel untranslated RNA (SR1) in *Bacillus subtilis*. *Mol. Microbiol.* 58 189–206. 10.1111/j.1365-2958.2005.04810.x 16164558

[B47] Marston A. L. Errington J. (1999). Selection of the midcell division site in *Bacillus subtilis* through MinD-dependent polar localization and activation of MinC. *Mol. Microbiol.* 33 84–96. 10.1046/j.1365-2958.1999.01450.x 10411726

[B48] Meniche X. Otten R. Siegrist M. S. Baer C. E. Murphy K. C. Bertozzi C. R. (2014). Subpolar addition of new cell wall is directed by DivIVA in mycobacteria. *Proc. Natl. Acad. Sci. U.S.A.* 111 E3243–E3251. 10.1073/pnas.1402158111 25049412 PMC4128124

[B49] Mierzejewska J. Jagura-Burdzy G. (2012). Prokaryotic ParA–ParB–parS system links bacterial chromosome segregation with the cell cycle. *Plasmid* 67 1–14. 10.1016/j.plasmid.2011.08.003 21924286

[B50] Miller M. D. Schwarzenbacher R. von Delft F. Abdubek P. Ambing E. Biorac T. (2004). Crystal structure of a tandem cystathionine-beta-synthase (CBS) domain protein (TM0935) from *Thermotoga maritima* at 1.*87* A resolution. *Proteins* 57 213–217. 10.1002/prot.20024 15326606

[B51] Mohamed J. A. Huang D. B. (2007). Biofilm formation by enterococci. *J. Med. Microbiol.* 56 1581–1588. 10.1099/jmm.0.47331-0 18033823

[B52] Muchová K. Kutejová E. Pribisová L. Wilkinson A. J. Barák I. (2002). *Bacillus subtilis* division protein DivIVA - screen for stable oligomer state conditions. *Acta Crystallogr. D Biol. Crystallogr.* 58 1542–1543. 10.1107/s0907444902014336 12351857

[B53] Mukherjee P. Sureka K. Datta P. Hossain T. Barik S. Das K. P. (2009). Novel role of Wag31 in protection of mycobacteria under oxidative stress. *Mol. Microbiol.* 73 103–119. 10.1111/j.1365-2958.2009.06750.x 19496931

[B54] Murray B. E. (1990). The life and times of the Enterococcus. *Clin. Microbiol. Rev.* 3 46–65. 10.1128/cmr.3.1.46-65.1990 2404568 PMC358140

[B55] Nguyen L. Scherr N. Gatfield J. Walburger A. Pieters J. Thompson C. J. (2007). Antigen 84, an effector of pleiomorphism in *Mycobacterium smegmatis*. *J. Bacteriol.* 189 7896–7910. 10.1128/JB.00726-07 17766411 PMC2168712

[B56] Nováková L. Bezoušková S. Pompach P. Špidlová P. Sasková L. Weiser J. (2010). Identification of multiple substrates of the StkP Ser/Thr protein kinase in *Streptococcus pneumoniae*. *J. Bacteriol.* 192 3629–3638. 10.1128/JB.01564-09 20453092 PMC2897338

[B57] Patrick J. E. Kearns D. B. (2008). MinJ (YvjD) is a topological determinant of cell division in *Bacillus subtilis*. *Mol. Microbiol.* 70 1166–1179. 10.1111/j.1365-2958.2008.06469.x 18976281

[B58] Paulsen I. T. Banerjei L. Myers G. S. A. Nelson K. E. Seshadri R. Read T. D. (2003). Role of mobile DNA in the evolution of vancomycin-resistant *Enterococcus faecalis*. *Science* 299 2071–2074. 10.1126/science.1080613 12663927

[B59] Perry S. E. Edwards D. H. (2006). The *Bacillus subtilis* DivIVA protein has a sporulation-specific proximity to Spo0J. *J. Bacteriol.* 188 6039–6043. 10.1128/JB.01750-05 16885474 PMC1540055

[B60] Pinho M. G. Errington J. (2004). A *divIVA* null mutant of *Staphylococcus aureus* undergoes normal cell division. *FEMS Microbiol. Lett.* 240 145–149. 10.1016/j.femsle.2004.09.038 15522501

[B61] Poyart C. Trieu-Cuot P. (1997). A broad-host-range mobilizable shuttle vector for the construction of transcriptional fusions to beta-galactosidase in Gram-positive bacteria. *FEMS Microbiol. Lett.* 156 193–198. 10.1111/j.1574-6968.1997.tb12726.x 9513264

[B62] Ragumani S. Sauder J. M. Burley S. K. Swaminathan S. (2010). Structural studies on cytosolic domain of magnesium transporter MgtE from *Enterococcus faecalis*. *Proteins* 78 487–491. 10.1002/prot.22585 19787770 PMC3221319

[B63] Ramirez-Arcos S. (2005). *Enterococcus faecalis divIVA:* an essential gene involved in cell division, cell growth and chromosome segregation. *Microbiology* 151 1381–1393. 10.1099/mic.0.27718-0 15870448

[B64] Ramirez-Arcos S. Szeto J. Beveridge T. Victor C. Francis F. Dillon J. (2001). Deletion of the cell-division inhibitor MinC results in lysis of *Neisseria gonorrhoeae*. *Microbiol. Read. Engl.* 147 225–237. 10.1099/00221287-147-1-225 11160816

[B65] Rigden M. D. Baier C. Ramirez-Arcos S. Liao M. Wang M. Dillon J.-A. R. (2008). Identification of the coiled-coil domains of *Enterococcus faecalis* DivIVA that mediate oligomerization and their importance for biological function. *J. Biochem. (Tokyo)* 144 63–76. 10.1093/jb/mvn044 18388125

[B66] Schumacher M. A. (2017). “Bacterial nucleoid occlusion: multiple mechanisms for preventing chromosome bisection during cell division,” in *Prokaryotic Cytoskeletons: Filamentous Protein Polymers Active in the Cytoplasm of Bacterial and Archaeal Cells Subcellular Biochemistry*, eds Löwe J. Amos L. A. (Cham: Springer International Publishing), 267–298. 10.1007/978-3-319-53047-5_9

[B67] Servant P. Le Coq D. Aymerich S. (2005). CcpN (YqzB), a novel regulator for CcpA-independent catabolite repression of *Bacillus subtilis* gluconeogenic genes. *Mol. Microbiol.* 55 1435–1451. 10.1111/j.1365-2958.2005.04473.x 15720552

[B68] Shepard B. D. Gilmore M. S. (1995). “Electroporation and efficient transformation of *Enterococcus faecalis* grown in high concentrations of glycine,” in *Electroporation Protocols for Microorganisms Methods in Molecular BiologyTM*, ed. Nickoloff J. A. (Totowa, NJ: Humana Press), 217–226. 10.1385/0-89603-310-4:217 7550738

[B69] Sieger B. Schubert K. Donovan C. Bramkamp M. (2013). The lipid II flippase RodA determines morphology and growth in Corynebacterium glutamicum. *Mol. Microbiol.* 90 966–982. 10.1111/mmi.12411 24118443

[B70] Sievert D. M. Ricks P. Edwards J. R. Schneider A. Patel J. Srinivasan A. (2013). Antimicrobial-resistant pathogens associated with healthcare-associated infections: summary of data reported to the national healthcare safety network at the centers for disease control and prevention, 2009-2010. *Infect. Control Hosp. Epidemiol.* 34 1–14. 10.1086/668770 23221186

[B71] Sigrist C. J. A. Cerutti L. de Castro E. Langendijk-Genevaux P. S. Bulliard V. Bairoch A. (2010). PROSITE, a protein domain database for functional characterization and annotation. *Nucleic Acids Res.* 38 D161–D166. 10.1093/nar/gkp885 19858104 PMC2808866

[B72] Tännler S. Fischer E. Le Coq D. Doan T. Jamet E. Sauer U. (2008). CcpN controls central carbon fluxes in *Bacillus subtilis*. *J. Bacteriol.* 190 6178–6187. 10.1128/JB.00552-08 18586936 PMC2546806

[B73] Torelli R. Cacaci M. Papi M. Paroni Sterbini F. Martini C. Posteraro B. (2017). Different effects of matrix degrading enzymes towards biofilms formed by *E. faecalis* and *E. faecium* clinical isolates. *Colloids Surf. B Biointerfaces* 158 349–355. 10.1016/j.colsurfb.2017.07.010 28715766

[B74] Wu L. J. Errington J. (2003). RacA and the Soj-Spo0J system combine to effect polar chromosome segregation in sporulating *Bacillus subtilis*: Chromosome segregation in B. *subtilis*. *Mol. Microbiol.* 49 1463–1475. 10.1046/j.1365-2958.2003.03643.x 12950914

[B75] Yogurtcu O. N. Johnson M. E. (2018). Cytosolic proteins can exploit membrane localization to trigger functional assembly. *PLoS Comput. Biol.* 14:e1006031. 10.1371/journal.pcbi.1006031 29505559 PMC5854442

[B76] Zou Y. Li Y. Dillon J.-A. R. (2017). The distinctive cell division interactome of *Neisseria gonorrhoeae*. *BMC Microbiol* 17:232. 10.1186/s12866-017-1140-1 29233095 PMC5727935

