# Supplementary material for: EF1025, a Hypothetical Protein From Enterococcus faecalis, Interacts With DivIVA and Affects Cell Length and Cell Shape
Source: Front Microbiol. 2020 Feb 12;11:83. doi: 10.3389/fmicb.2020.00083 (PMC7028823; doi:10.3389/fmicb.2020.00083)
Supplement: Supplementary file 1 [file Data_Sheet_1.docx]

**Strains, plasmids and growth conditions**

Plasmid DNA was purified using Plasmid Mini-prep or Plasmid Midi-prep Kits (Qiagen Inc., ON, Canada). Reading frame conservation and gene integrity of all plasmids was confirmed by DNA sequencing [Core DNA Synthesis and Sequencing Facility, Centre for Research in Biopharmaceuticals and Biotechnology, University of Ottawa, (UOCDSSF), the Plant Biotechnology Institute (PBI), National Research Council of Canada, Saskatoon, Saskatchewan] or Eurofins Canada. Primers (Table S3) were synthesized at the UOCDSSF and Invitrogen (Thermo Scientific; Waltham, MA), and were used for PCR and DNA sequencing reactions. PCR reactions were carried out using Q5 DNA polymerase (New England BioLabs Ltd., ON, Canada) in a Perkin Elmer GenAmp PCR System 9600 Thermocycler (Perkin Elmer, Inc., Woodbridge, ON, USA).

**Cloning and screening an *E. faecalis* genomic DNA library by Y2H assay**

An *E. faecalis* JH2-2 genomic DNA library was created in the Y2H system (Clontech) using the vector pGAD424 of the Clontech Matchmaker GAL4 Two-Hybrid System (Clontech) (Table S2C). *E. faecalis* JH2-2 genomic DNA was prepared using the Wizard Genomic DNA Purification Kit according to the manufacturer’s instructions (Promega, Madison, WI USA). Approximately 10 µg genomic DNA was partially digested with Sau3AI and size-fractionated by agarose gel electrophoresis. DNA fragments ranging between 0.2- to 1.5-kb were excised from the gel and purified using PCR Purification Kit (Qiagen). Purified DNA fragments were then ligated to pre-cleaved *Bam*HI-pGAD424. The ligation mixture was transformed into *E. coli* DH5α competent cells and transformants were selected on LB plates supplemented with Amp 100 µg/ml (LB-Amp). Colonies were harvested by washing the plates with LB-Amp broth. Approximately 1×10^5^ colonies were collected in 50 ml LB-Amp broth which was incubated at 37°C for 2 hrs, followed by centrifugation to collect pelleted cells. Plasmid DNA was purified using Midi-prep Kit (Qiagen) and was named pGAD424-Lib (Table S2C). Colony counts were estimated by serially diluting an aliquot of the cell suspension in LB-Amp broth.

To determine the ratio of colonies harbouring a plasmid with an inserted DNA fragment and sizes of the inserts, 30 individual colonies were randomly selected from the original library and were sub-cultured on LB-Amp broth. Plasmid DNA was purified and double digested with EcoRI/BglII followed by electrophoresis on 1% agarose gels. 77% (23/30) of the recombinant clones carried inserts of sizes ranging between ~350 bp to ~2 kb. To determine the quality of the library, an aliquot of the purified library plasmid DNA (pGAD424-Lib) or the parental vector pGAD424 DNA was digested with SnaBI/PstI. The digested library DNA (pGAD424-Lib) exhibited DNA fragments of various sizes that were bigger than 1.5 kb, indicating that the majority of the library plasmid DNA carried inserts (data not shown).

To screen the library, the previously constructed plasmid pSRBD-Div was used to express the bait protein, DivIVA_Ef_ (Table S2C; (Ramirez-Arcos 2005)). Plasmids pSRBD-Div and pGAD424-Lib were co-transformed into *S. cerevisiae* SFY526 according to the manufacturer’s instructions (Clontech). Transformants were selected on complete synthetic medium lacking leucine and tryptophan (SD-leu-trp) (Clontech). After 3-4 days of incubation at 30°C, blue-coloured clones were screened in the presence of 5-Bromo4-chloro-3-indolyl-β-D-galactopyranoside (X-Gal, Sigma-Aldrich; St. Louis, MS) by a colony–lift filter assay (Clontech). Positive clones were streaked on SD-leu-trp medium plates (Clontech). A spectrophotometric assay for β-galactosidase activity, using the substrate o-nitrophenyl β-_D_-galactopyranoside (ONPG liquid assays), was performed to confirm the results of colony-lift assay (Ramirez-Arcos 2005). Transformation efficiency was monitored by plating 50 µL of diluted transformants on SD-leu-trp medium plates followed by counting the number of colonies produced.

In a positive clone, pGAD424-Lib plasmid was separated from a pSRBD-Div by sub-culturing the yeast cells of the positive clone in SD-leu-trp broth for 2-4 days at 30°C. Cells were harvested by centrifugation and the cell pellet was re-suspended in 250 μL of Qiagen buffer P1 (Qiagen plasmid mini-prep kit) with 10 μL glass beads (Sigma), followed by vigorous vortexing for 3 min. P2 buffer (250 µL, Qiagen) was added to the lysate, and plasmid DNA was purified. To isolate plasmid pGAD424-Lib, the aforementioned purified plasmid DNA was transformed into *E. coli* DH5α cells and the resulting *E. coli* colonies were examined for plasmid content in a cracking assay (Ramirez-Arcos 2005). The size of released supercoiled plasmid DNA was determined by electrophoresis on 1 % agarose gels. The difference in the size of pSRBD-Div (6.2 kb) and pGAD424-Lib (≥6.6 kb) allowed easier separation from each other. The plasmid of interest (i.e. pGAD424-Lib) was then purified from *E. coli* transformants and analyzed by restriction endonuclease digestion with EcoRI/PstI. Purified plasmid DNA was sequenced at the UOCDSSF using primers AD424F and AD424R (Supplementary Materials, Table S3C) to generate DNA sequences of the inserts in pGAD424-Lib for bioinformatic identification of the discovered genes.

**Reverse transcriptase PCR (RT-PCR)/qPCR**

Total RNA from *E. faecalis* JH2-2 and MJ26 was isolated using the Qiagen RNeasy Total RNA kit (Qiagen) for RT-PCR assay which was performed as previously described (Fadda et al. 2003). cDNA was created from total isolated RNA by incubating ~0.1 µg RNA, 0.5 unit reverse transcriptase (Promega) and 2 µl random primer mix at 42°C for 30 min. This cDNA was used to amplify *EF1026* from JH2-2 and MJ26 using primers EF26aF/R, EF26bF/R (Table S3E). The housekeeping gene, *gdh* (encoding glucose dehydrogenase) was used as a positive control and was PCR amplified using primers HKaF/R, HKbF/R (Table S3E). PCR amplification of genomic DNA using primers EF26aF/R served as a positive control whereas PCR amplification of total RNA using primers EF26aF/R served as a negative control. PCR products were separated by electrophoresis on 1.5% agarose gel for further analysis. For qPCR, cDNA from *E. faecalis* JH2-2 was used to create standards using primers EF26aF/R (Table S3E) and was used to identify EF1026 levels in *E. faecalis* MJ26. Each reaction was performed in triplicate and contained 2X SYBR-Green master mix (Cat # 4472912, Life Technologies Inc.), 0.25 µL of each primer (10 µM), 1 µL of DNA (50 ng/µL), and 3.5 µL PCR-grade water in a total 10 µL reaction volume.

**Expression of *EF1025* in *E. coli* PB103**

To express *EF1025* in *E. coli* PB103, *EF1025* was PCR-amplified from *E. faecalis* JH2-2 and cloned into pUC18 (Amersham), resulting in plasmid pUCHisEF1025 (Table S2F). For controls, *prgX*, a transcriptional regulator of itself and PrgB (cell wall aggregation substance) (Bhatty et al., 2015; Bae et al., 2000), was PCR-amplified from pSR-X (Table S2F; Bae et al., 2000; Rigden et al., 2008) and cloned into pUC18, resulting in plasmid pUCHisPrgx, which encodes 6×His tagged PrgX (Table S2F). Each plasmid was individually transformed into *E. coli* PB103 and transformants were selected on LB medium supplemented with Amp100 creating strains *E. coli* PB MK23 and *E. coli* PB MK25, respectively (Table S1). Expression of 6×His-EF1025 or 6×His-PrgX was determined by Western blot assays using anti-6×His monoclonal antibodies (Biorad).

**Atomic force microscopy**

For atomic force microscopy, cell suspensions from overnight grown cultures of *E. faecalis* were deposited onto Cell-Tak (LifeTechnologies) coated coverslips for 30 min, fixed with formalin, and air-dried prior to AFM imaging (Bhat et al., 2015). Samples were imaged with silicon nitride cantilevers (HYDRA6R-200NG; Nanosensors, Neuchatel, Switzerland) with calibrated spring constants ranging from 0.03 to 0.062 N/m. QI™ images and force curves (JPK software) at each pixel of a 128 x 128 raster scan were collected using a Z-length of 0.926 um and a scan rate of 95 um/s. Surface roughness was calculated according to Bhat et al. (2015) from multiple 200 x 200 nm squares along the centre of the cell from QI™ height images for at least 10 cells each from three biological replicates.

The morphology of *E. coli* PB103 harbouring pUCHisEF1025 was ascertained using an Olympus BX61 microscope (Olympus Canada Inc.), as described previously (Ramirez-Arcos et al., 2001). At least 30 fields were examined each containing a minimum of 40 cells.

**Statistical analysis**

All studies were conducted in triplicates and Microsoft Excel was used for statistical analysis unless otherwise indicated. The results were reported as mean ± standard deviation (SD), differences assessed using a two-tailed unpaired t-test and ANOVA for which p<0.05 was considered statistically significant.
