# Supplementary material for: EF1025, a Hypothetical Protein From Enterococcus faecalis, Interacts With DivIVA and Affects Cell Length and Cell Shape
Source: Front Microbiol. 2020 Feb 12;11:83. doi: 10.3389/fmicb.2020.00083 (PMC7028823; doi:10.3389/fmicb.2020.00083)
Supplement: Supplementary file 2 [file Data_Sheet_2.docx]

Table S1. Bacterial and yeast strains used in this study

| Strains | Relevant characteristics | Resources or references |
| --- | --- | --- |
| *Escherichia coli* XL1-Blue | *recA1 endA1 gyrA96 thi-1 hsdR17 supE44 relA1 lac* [F´ *proAB lacI*q*Z*_*M15*Tn*10* (Tetr)] | Strategene |
| *E. coli* DH5α | *endA*1 hsdr17 (r_k_^-^m_k_^+^) *supE*44 *thi*-1 *recA*1 *gyrA*96 *relA*1 Δ(*arg*F-*lac*ZYA) *U*169 *deoR* [ø80d *lac* Δ(*lacZ*) M15) | Gibco-BRL |
| *E. coli* C41 (DE3) | Fˉ*ompT hsdS_B_ (r_B-_m_B-_) gal dcm Δ(srl-recA)* 306*::*Tn*10* *(tet^R^) (DE3)* | Miroux *et al.*, 1996 |
| *E. coli* PB103 | *dadR1 trpE61 trpA62 tna-5 purB^+^* | de Boer *et al.,* 1988 |
| *E. coli* R721 | 71/18 *glpT*::O-P434/P22*lacZ* | Di Lallo *et al.*, 2001, 2003 |
| *Enterococcus faecalis* JH2-2 | Rif^R^, Fus^R^; plasmid free | Jacob & Hobbs, 1974 |
| *Sacharomyces cerevisiae* SFY526 | *MATa ura3-52 his3-200 ade2-101 lys2-801 trp1-901 leu2-3 112 can^r^ gal4-542 gal80-538 URA3::GAL1_UAS-_ GAL1_TATA_ –lacZ* | Clontech Laboratories, CA |
| *E. faecalis* MK0 | *E. faecalis* JH2-2 carrying pMSP3545A | This study |
| *Enterococcus faecalis* MK23 | *E. faecalis* JH2-2 carrying pMSPEF1025A (P*_EF1025_*-*EF1025*) for expressing EF1025 *in trans* under its native promoter. Ery^R^ (125 µg/mL) | This study |
| *Enterococcus faecalis* MK24 | *E. faecalis* JH2-2 carrying pMSPEF1025-flag (P*_EF1025_*-*EF1025-flag*) for expressing EF1025-flag *in trans* under its native promoter. Ery^R^ (125 µg/mL) | This study |
| *E. faecalis* MJ26 | Derived from *E. faecalis* JH2-2 with insertionally inactivated *EF1025* (*EF1025::kan^R^*). *E. faecalis* MJ26 carried pMSPEF1025-Pro (P*_EF1025_*-*EF1025*) for expressing EF1025 *in trans* under its native promoter. Kan^R^ (500 µg/mL) and Ery^R^ (125 µg/mL) | This study |
| *E. faecalis* MK12 | Derived from *E. faecalis* JH2-2 with deletion of *EF1025* (Δ*EF1025::cat^R^*). *E. faecalis* MK12 carried pMSPEF1025A (P*_EF1025_*-*EF1025*) for expressing *EF1025* *in trans* under its native promoter. Cat^R^ (5 µg/mL) and Ery^R^ (125 µg/mL). | This study |
| *E. coli* PB MK23 | Derived from *E. coli* PB103 for overexpressing *EF1025* using pUCHisEF1025. Amp^R^ (100 µg/mL) | This Study |
| *E. coli* PB MK25 | Derived from *E. coli* PB103 for overexpressing *prgX* using pUCHisPrgx. Amp^R^ (100 µg/mL). | This Study |
|  | | |

Table S2. Plasmids used in this study

| Plasmid | Relevant characteristics | Sources or references |
| --- | --- | --- |
| (A) Plasmids for bacterial two-hybrid assays | |  |
| pcI434 | Kan^R^, bacterial two-hybrid vector | Di Lallo *et al.*, 2001 |
| pcIp22 | Amp^R^, bacterial two-hybrid vector | Di Lallo *et al.*, 2001 |
| pcIp22-L | pcI_P22_ derivative carrying a linker with multiple cloning sites | This study |
| pcI434-L | pcI_434_ derivative carrying a linker with multiple cloning sites | This study |
| pdivIVA22 | pcI_P22_ derivative carrying *E. faecalis* *divIVA* | This study |
| pdivIVA434 | pcI_434_ derivative carrying *E. faecalis* *divIVA* | This study |
| pEF1025434 | pcI_P434_L derivative carrying *EF1025* | This study |
| p22CBS1CBS2 | pcI_P22_L derivative carrying *EF1025* fragment coding AA80-204 | This study |
| p434CBS1CBS2 | pcI_434_L derivative carrying *EF1025* fragment coding AA80-204 | This study |
|  |  |  |
| (B) Plasmids for GST pull-down assays and 6xHis tagged protein expression | |  |
| pGEX-2T | Amp^R^ P_lac_*::gst* | Amersham Bioscience |
| pGST-Div | Amp^R^ P_lac_*::gst*, GST-DivIVA_Ef_ | This study |
| pET30a(+) | Kan^R^ P_T7_::*6xhis* | Novagen |
| pETEF1025 | Kan^R^ P_T7_, 6xHis-EF1025 | This study |
| pETEF1025CBS12 | Kan^R^ P_T7_, 6xHis-EF1025 with CBS1 and CBS2 domains | This study |
|  |  |  |
| (C) Plasmids for EF1025 self-interaction studies | |  |
| pGAD424 | Amp^R^ P_ADH1_*::gal4* (AD) | Clonetech, CA |
| pGBT9 | Amp^R^ P_ADH1_*::gal4* (DBD) | Clonetech, CA |
| pGADEF1025CBS12 | Amp^R^ P_ADH1_*::gal4* (AD), AD-EF1025 with CBS1 and CBS2 domains (AA80-204) | This study |
| pGBDEF1025CBS12 | Amp^R^ P_ADH1_*::gal4* (DBD), DBD- EF1025 with CBS1 and CBS2 domains (AA80-204) | This study |
| pGADEF1025NCBS1 | Amp^R^ P_ADH1_*::gal4* (AD), AD-EF1025 with N-terminal and CBS1 domains (AA1-137) | This study |
| pGBDEF1025NCBS1 | Amp^R^ P_ADH1_*::gal4* (DBD), DBD-EF1025 with N-terminal and CBS1 domains (AA1-137) | This study |

Table S2. Plasmids used in this study (Contd.)

| Plasmid | Relevant characteristics | Sources or references |
| --- | --- | --- |
| pGADEF1025CBS2 | Amp^R^ P_ADH1_*::gal4* (AD), AD-EF1025 with CBS2 domain (AA137-204) | This study |
| pGBDEF1025CBS2 | Amp^R^ P_ADH1_*::gal4* (DBD), DBD- EF1025 with CBS2 domain (AA137-204) | This study |
| pGADEF1025-N | Amp^R^ P_ADH1_*::gal4* (AD), AD-EF1025 with N-terminal domain (AA1-50) | This study |
| pGBDEF1025-N | Amp^R^ P_ADH1_*::gal4* (DBD), DBD- EF1025 with N-terminal domain (AA1-50) | This study |
| pGADEF1025 | Amp^R^ P_ADH1_*::gal4* (AD), AD-EF1025 (AA1-209) | This study |
| pGBDEF1025 | Amp^R^ P_ADH1_*::gal4* (DBD), DBD-EF1025 (AA1-209) | This study |
| pSRBD-Div | Amp^R^ P_ADH1_*::gal4* (DBD), DBD-DivIVA_Ef_ | (Ramirez-Arcos, 2005) |
| pGAD424-Lib | *E.faecalis* genomic DNA library constructed in pGAD424 vector | This study |
|  |  |  |
| (D) Plasmids for construction of an *EF1025* insertion or deletion strain and plasmids to overexpress *EF1025* in *E. faecalis* JH2-2 | | |
| pMSP3545 | Ery^R^ P_nisA_*::nisA* | Callegan *et al.*, 1999 |
| pcDNA3.1(+) | Amp^R^ Neo^R^, P_lac_, P_SV40_ and P_T7_*::flag* | Invitrogen |
| pMSP3545A | Ery^R^, Amp^R^, P_nisA_*::nisA* | This study |
| pMSPEF1025A | Ery^R^, Amp^R^ P_EF1025_*::EF1025* for  *EF1025*expression under its native promoter | This study |
| pMSPEF1025-flag | Ery^R^, Amp^R^ P_EF1025_*::EF1025* for *EF1025* expression under its native promoter with flag tag on C-terminus | This study |
| pMSPEF1025-pro | Ery^R^ P_mljd_*::mljd1* for *EF1025* expression under its native promoter | This study |
| p3ERMEF1025::Kan | p3ERM Δ*Hind*III, *EF1025::Kan* | This study |
| p3ERMΔEF1025::Cat | p3ERM Δ*Hind*III, Δ*EF1025::Cat* | This study |
| pUC18 | Amp^R^ P_lac_*::lacZ* | Amersham Biosciences |
| pUCEF1025-N | N-terminus of EF1025 ligated in pUC18 | This study |
| pTCV-lac | Kan^R^:*:lacZ* | Poyart & Trieu-Cuot, 1997 |
| pUCEF1025-N-Kan | N-*EF1025* (5’)-*kan^R^* | This study |
| pUCEF1025::Kan | *EF1025::kan^R^* | This study |
| pLEMO | Cat^R^, P_T7_, pACYC184 derivative carrying *lysY* | New England Biolabs |
|  |  |  |
| (E) Plasmids for heterologous expression of *EF1025* in *E. coli* | | |
| pUCHisEF1025 | Amp^R^ P_lac_, 6xHis-EF1025 | This study |
| pSR-X | Amp^R^ P_lac_, PrgX | This study |
| pUCHisPrgx | Amp^R^ P_lac_, 6xHis-Prgx | This study |

Table S3. Primers used in this study

| **Primer** | **Sequence (5’ to 3’)** |
| --- | --- |
| (A) Primers for B2H experiments | |
| EF1025-F | GCGTCGAC TTATCTGTTTTGTGCG |
| EF1025-R | GCGGATCCCTACGTAATATAGGTTAAAATTTTCGT |
| EF1025C-F | GCGTCGACGGAGATCATGAGTCCACCA |
| EF1025C-R | GCGGATCCCTACGTAATATAGGTTAAAATTTTCGT |
| CBdivIVA-F | GCGTCGACTATGGCATTAAC |
| CBdivIVA-R | GCGGATCCCTATTTTGATTC |
|  |  |
| (B) Primers for GST pull-down assays | |
| IVA-5 | GCGCGGATCCATGGCATTAACTCCATTAGA |
| IVA-11 | GCGCGAATTCTTACTATTTTGATTCTTCTTCAA |
| EF1025F-F | CGCTTAAGTTATCTGTTTTGTGCG |
| EF1025F-R | CGGGATCCATGAAATTAAGTAAACG |
| EF1025-CF | CGCGGATCCCCACCATTGATGGTTGCCCAAGAC |
| EF1025-CR | GCCCTCGAGCCCTTATCTGTTTTGTGCGGCTTC |
|  |  |
| (C) Primers for EF1025 self-interaction studies and other Y2H assays | |
| AD424F | ACCACTACAATGGATGAT |
| AD424R | ACAGTTGAAGTGAACTTG C |
| CBSDPF | GCCGGAATTCATGAAATTAAGTAAACG AC |
| CBSDPR2 | AAACTGCAGTTATCTGTTTTGCGGC |
| CBSAA80F | CGGGATCCATGAGTCCACCAT TG |
| CBSAA137R | AAACTGCAGTTAATTTAAAGAGGC |
| CBSAA137F | CGGAATTCAATACAAATATTGATGGC |
| DEORR | AAACTGCAGTTAAACTTTCGGACTTGC |
| AD424F | ACCACTACAATGGATGAT |
| AD424R | ACAGTTGAAGTGAACTTG C |

Table S3. Primers used in this study (Contd.)

| **Primer** | **Sequence (5’ to 3’)** |
| --- | --- |
| (D) Primers for construction of an *EF1025* knockout strain and plasmids to overexpress *EF1025* in *E. faecalis* JH2-2 | |
| AmpF | GGAGTCTAGAGCTACCATGGATCCGTGCGCGGAACCCCTATTTG |
| AmpR | GAACGAGATCTGTCTGACGCTCAGTGGAACG |
| LinkA | GGTGTCAACGATATCCTCC |
| LinkB | AATTGGAGGATATCGTTGACACCTTC |
| EF1025npF | GAGCCCATGGCGTGACCTCCGTTTAATATGTG |
| EF1025npR | GGGTCTAGATTAAGCTCCCTTATCTGTTTTGTG |
| CBSDPF | GCCGGAATTCATGAAATTAAGTAAACG AC |
| CBS55-R-Hind | CCCAAGCTTAACTTTCGGACTTGC |
| KanF | CCCAAGCTTGTGGTTTCAAAATCG |
| KanR | TCCCCCGGGTTAGGTACTAAAACA |
| CBS55-F-Sma | TCCCCCGGGGCAAGTCCGAAAGTTG |
| EF1025-R-BamHI | CGGGATCCTTATCTGTTTTGTGCGGC |
| Mut-F | CTCTTTACCTTCATTGTGTG |
| ProF | AACTGCAGCAAAATTTCTGATTGTAAGTG |
| CBSDPR | AAACTGCAGTTATCTGTTTTGCGGC |
| ppdKF | GAGGGATCCAGCACCGCTGCGAACGGAAACTAAG |
| ppdKR | CCAGTGATTTTTTTCTCCATCATTTCCTCCTCAATTCCTC |
| 1026F | GAGTGGCAGGGCGGGGCGTAAGGGAGCTTAATTATGAAAAAAGAG |
| 1026R | GAGGAATTCTACATACTGACTGGCGTCTTTGAGG |
| CatF | GAGGAATTGAGGAGGAAATGATGGAGAAAAAAATCACTGGATATAC |
| CatR | CTTTTTTCATAATTAAGCTCCCTTACGCCCCGCCCTGCCACTC |
| FlagF | GATCTTTATAATCACCGTCATGGTCTTTGTAGTCG |
| FlagR | GAGATCTAGACTACTTGTCATCGTCATCCTTG |

Table S3. Primers used in this study (Contd.)

| **Primer** | | | **Sequence (5’ to 3’)** |
| --- | --- | --- | --- |
| (E) Primers used for RT-PCR | | | |
| EF25aF | | CGCATTTCGGACATACTAGC | |
| EF25aR | | TTGGGCAACCATCAATGGTG | |
| EF26aF | | TCAAGCGAAAGCCGGAGTAG | |
| EF26aR | | ACTGACTGGCGTCTTTGAGG | |
| EF26bF | | CAGTCGGTTGGCTTCCTTAG | |
| EF26bR | | CACTGGGATGCCATACTTCG | |
| HKaF | | TGGTGCAGCTACGGGTTTAG | |
| HKaR | | CTTTAGGCAGCTCACCGACA | |
| HKbF | | CTGGTGCAGCTACGGGTTTA | |
| HKbR | | GCTCACCGACATAGTCAGCA | |
| (F) Primers used for construction of plasmids to express *EF1025* in *E. coli* PB103 | | |  |
| HisEF1025F3 | | CGGAATTCGCACCATCATCATCATCATATGAA |  |
| EF1025-R-BH | | CGGGATCCTTATCTGTTTTGTGCGGC |  |
| HisPrgxF2 | | CGGAATTCGCACCATCATCATCATCATATGAC |  |
| PrgxR2 | | GCTCTAGATTAGTTTAAGATAGGTTC |  |
