# Supplementary material for: EF1025, a Hypothetical Protein From Enterococcus faecalis, Interacts With DivIVA and Affects Cell Length and Cell Shape
Source: Front Microbiol. 2020 Feb 12;11:83. doi: 10.3389/fmicb.2020.00083 (PMC7028823; doi:10.3389/fmicb.2020.00083)

Figure S1. Western blot exhibiting specificity of anti-DivIVA_Ef_ and anti-EF1025 antibody for DivIVA_Ef_ and EF1025. An *E. faecalis* whole cell lysate was probed with anti-DivIVA_Ef_ (Lanes 1-4), and anti-EF1025 (Lanes 5-8). A protein ladder confirmed the presence of protein bands of sizes corresponding to DivIVA_Ef_ (40 kDa) or EF1025 (27 kDa).


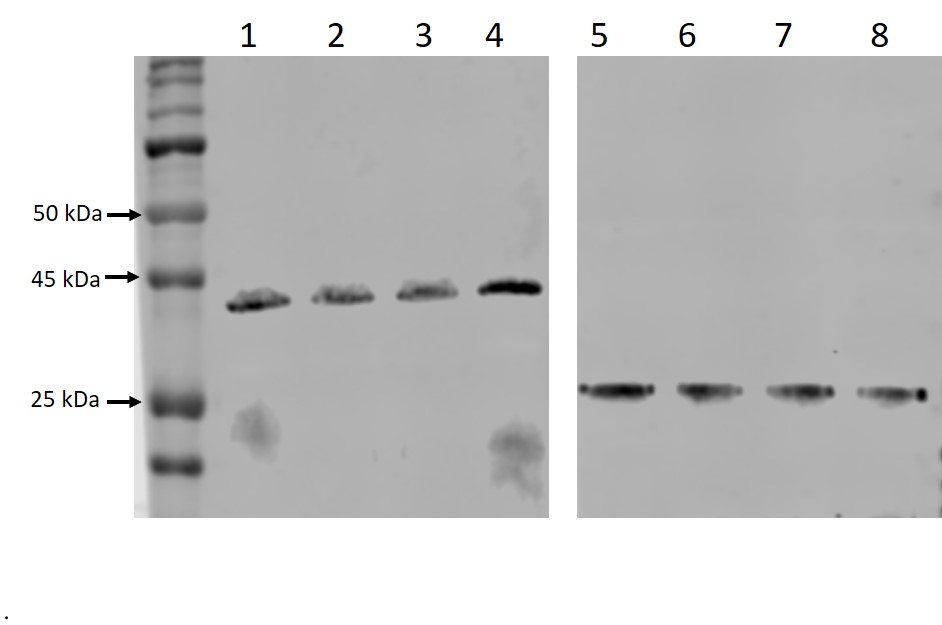


Figure S2*.* Light scattering (LS) data and measured molar mass for EF1025 (under denaturing conditions). Separated by SEC and detected using the μDAWN and UT-rEX (red) detected with the **Wyatt** TREOS and Optilab T-rEX (blue). The plot shows the chromatograms as a function of elution time. The average molecular weight calculated was 222 kDa for the complex. Black line shows aggregation profile of the protein.


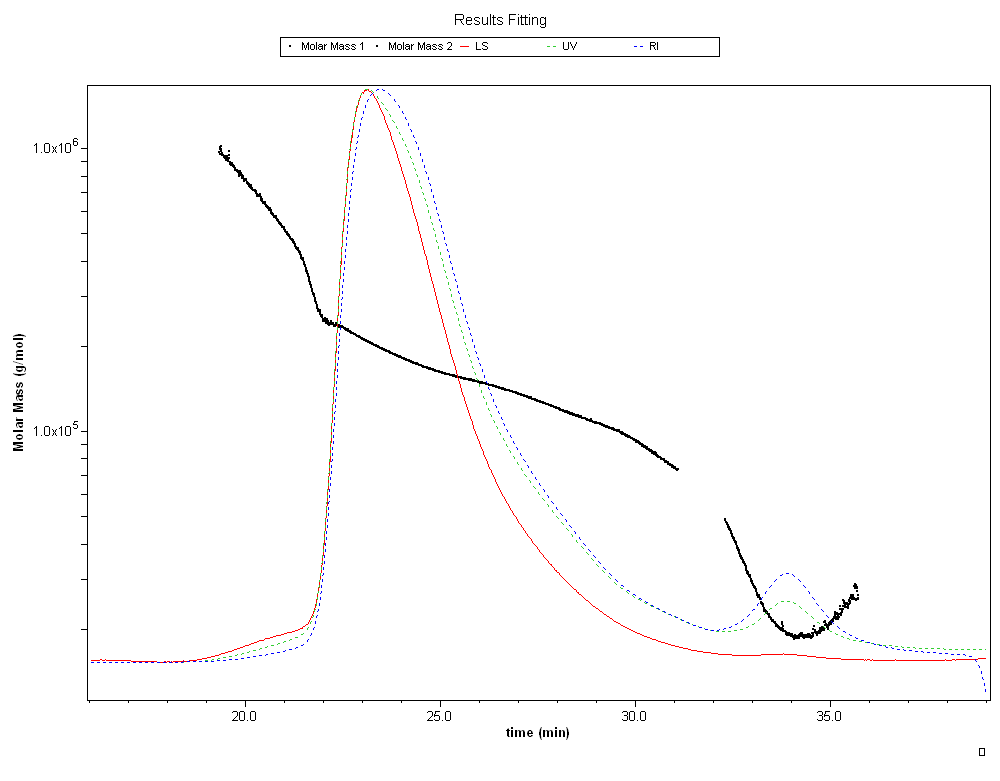


Figure S3. EF1025CBS12 interacts with DivIVA_Ef_ in GST pull-down assay. Shown is a Western blot probed with an anti-6xHis EF1025 monoclonal antibody. Lane 1: Protein Ladder; Lane 2: GST bound beads; Lane 3: GST-DivIVA_Ef_ bound beads; Lane 5: *E. faecalis* extracts representing 10% input of EF1025CBS12.


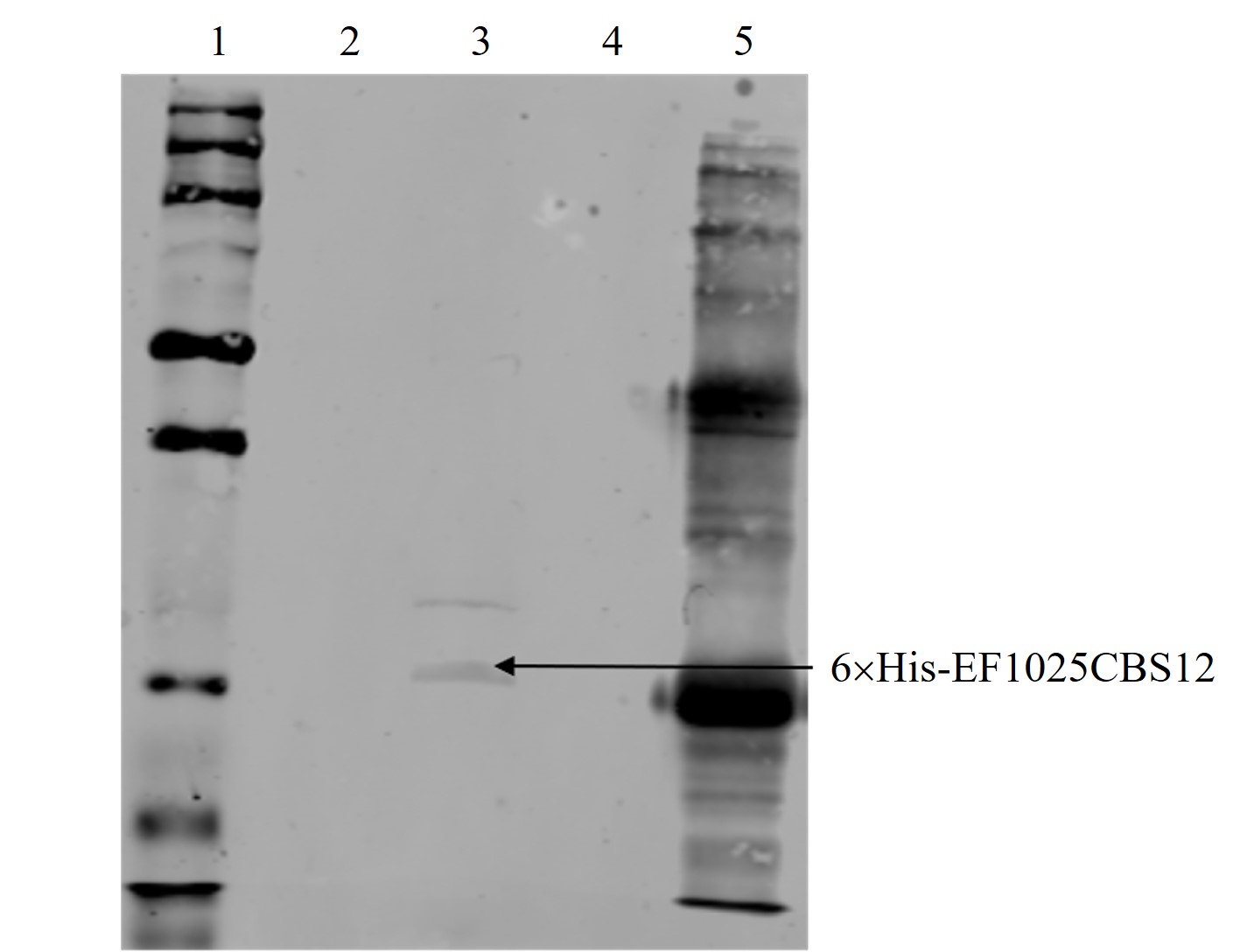


Figure S4. Confirmation of Kan^R^ cassette insertion in *EF1025* in *E faecalis* MJ26. **(A)** Schematic presentation of genomic insertional inactivation of *EF1025* in *E. faecalis*. A *kan^R^* cassette was inserted at the nt151 position of *mljd1*. Arrows indicated primers used for PCR amplification to confirm Kan^R^ insertion in the *E. faecalis* genomic DNA, **(B)** PCR confirmation of insertional mutation. PCR was performed on *E. faecalis* MJ26 genomic DNA using primer pairs Mut-F/Kan-R (Lane 1- 1300 bp), Kan-F/Kan-R (Lane 2- 930 bp), Pro-F/Kan-R (Lane 3- 1500 bp), Kan-F/CBSDPR (Lane 4- 1409 bp) and CBSDPF/CBSDPR (Lane 5- 1560 bp and 630 bp). M: 1kb plus DNA ladder. Presence of wild type *EF1025* was due to the presence of co-transformed plasmid pMSPEF1025-Pro (Lane 5). Lane 6: cropped lane from same gel with amplified wild type *EF1025*.

A.
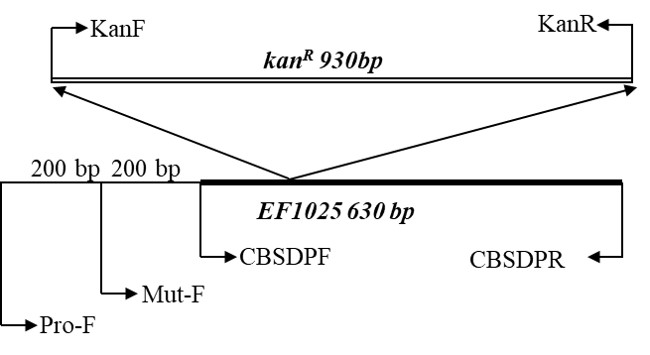


B.
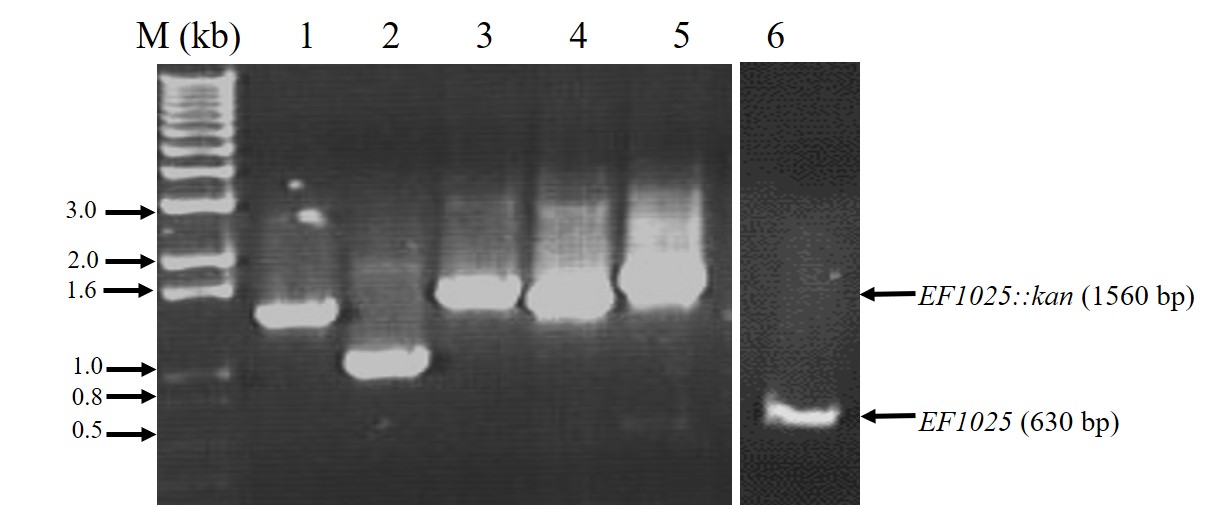


Figure S5. *E. faecalis* MJ26 and MK12 grew slower than *E. faecalis* JH2-2 cells. Viability curve of *E. faecalis* MJ26 cells. Growth was measured by OD at 600nm. *E. faecalis* MJ26 cells were subcultured on BHI containing appropriate antibiotics. Samples were withdrawn for plating every 2 hours. X- axis: Viable counts (CFU/ml), Y- axis- time (hours). X marked line- *E. faecalis* MK12; Open triangle line- *E. faecalis* JH2-2; Closed triangle line- *E. faecalis* MJ26.

Figure S6. RT-PCR of EF1026 in *E. faecalis* JH-2-2 and MJ26 showing absence of polar effect. PCR amplified products corresponding to EF1026 from JH-2-2 and MJ26. Lanes 1 and 2: EF_1026 from *E. faecalis* JH-2-2, Lanes 3 and 4: *gdh* from *E. faecalis* JH-2-2, Lane 5: negative control E*. faecalis* JH-2-2 with no reverse transcriptase; Lanes 6 and 7: EF_1026 from *E. faecalis* MJ26, Lanes 8 and 9: *gdh* from *E. faecalis* MJ26, Lane 10: negative control from E*. faecalis* MJ26 with no reverse transcriptase. *gdh*- glucose dehydrogenase (housekeeping gene).


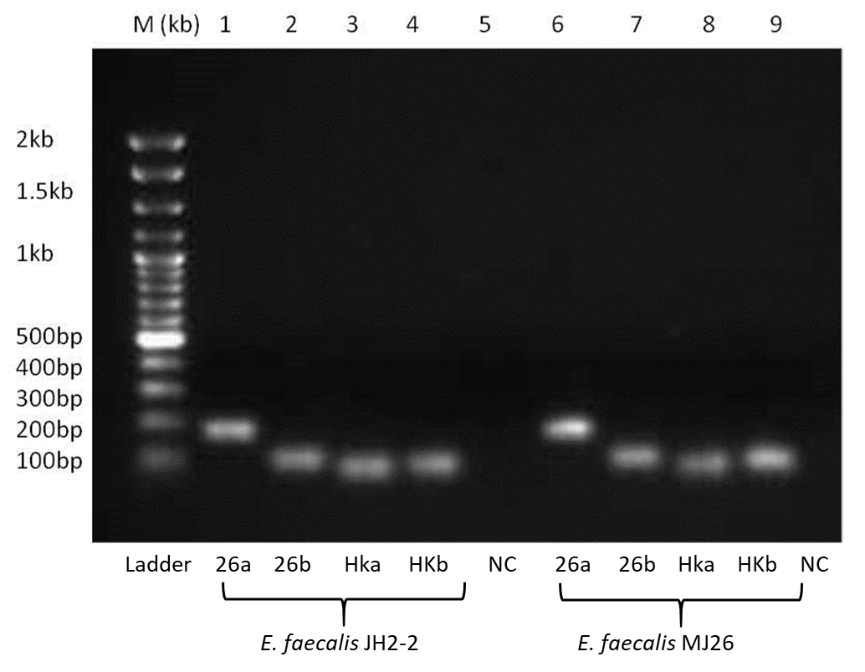


**Figure S7. *E. faecalis* MK12 cells exhibit larger aggregates than *E. faecalis* JH2-2.**Representative AFM error images of *E. faecalis* JH2-2 **(A)** and *E. faecalis* MK12 **(B)**collected in QI mode with a resolution of 128×128 pixels per image. Both JH2-2 and MK12 form relatively frequent cell aggregates that are larger for MK12. Since these clusters had irregular shapes and cell numbers, sizes could not be accurately estimated. Bar scale (5 µm) indicated at the bottom right corner of each image.


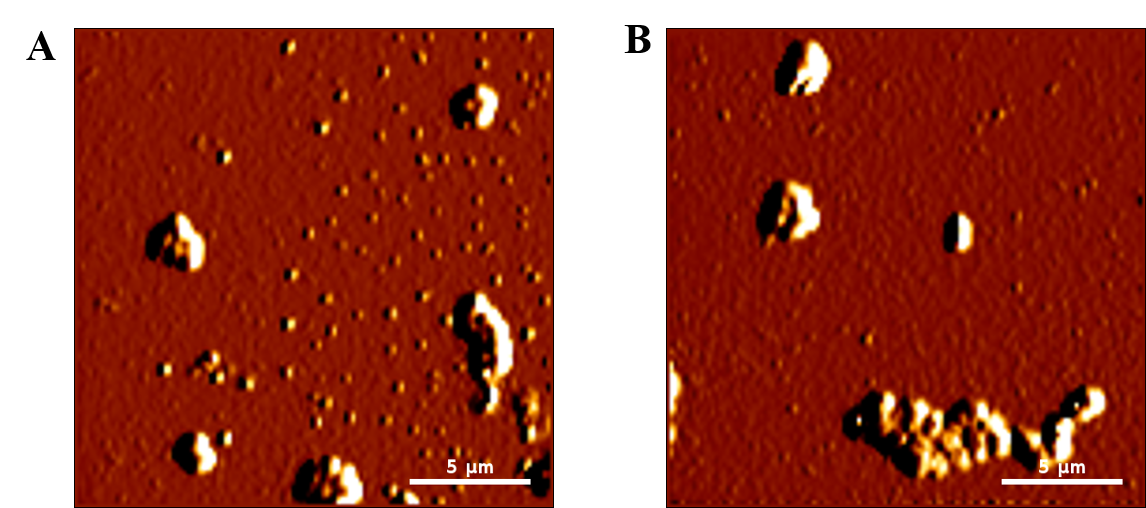


Figure S8. Shown are (A) Western blot probed with anti-Flag antibody to detect the presence of EF1025-flag in *E. faecalis* MK24. (B) Representative Western blot probed with anti-EF1025 to detect the presence of EF1025. Whole cell extract from: Lane 1: *E. faecalis* JH2-2; Lane 2: *E. faecalis* MK23; and Lane 3: *E. faecalis* MK24. (C) Densitometric quantification of band intensities corresponding to EF1025 from strains *E. faecalis* JH2-2, *E. faecalis* MK23, and *E. faecalis* MK24.


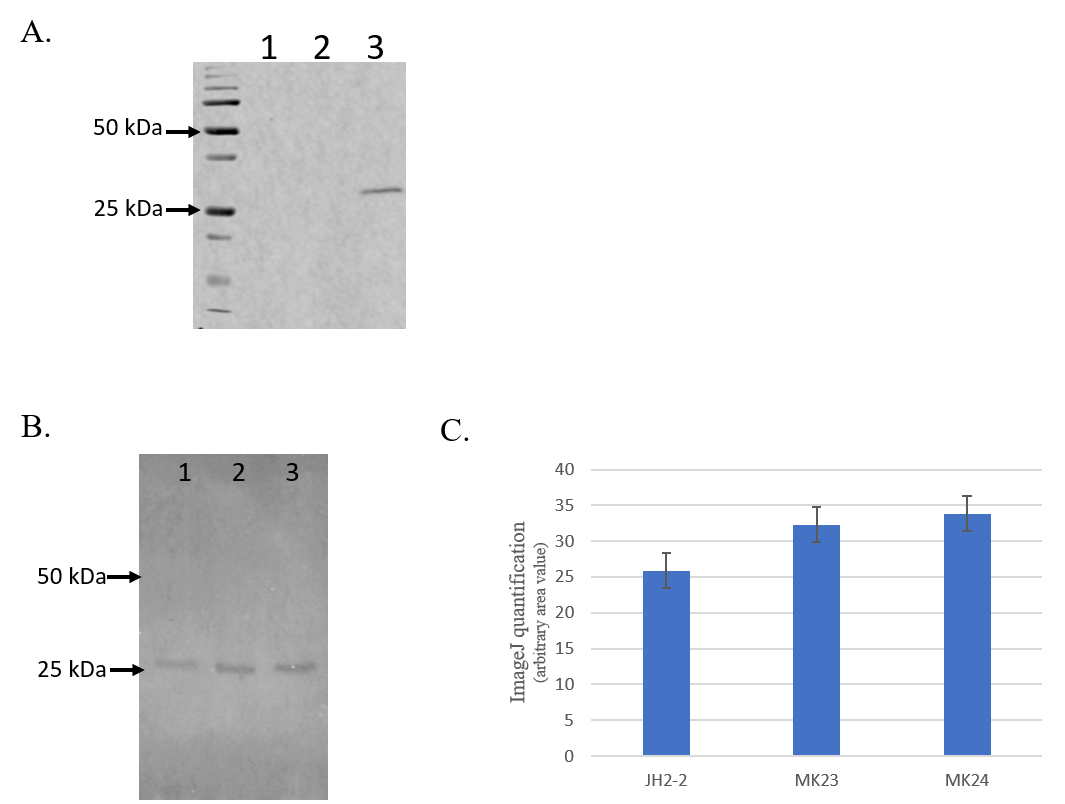


Figure S9. Overexpression of *EF1025* in *E. coli* PB103 leads to severe cell elongation. Phase contrast microscopy of *E. coli* PB103 cells. (**A)** *E. coli* PB103 cells; (**B)** filamentous *E. coli* PB MK23 (>15 µm) cells transformed with pUCHisEF1025, and **(C)** *E. coli* PB MK25 overexpressing *prgX_Ef_*. Scale bars represent 25 μm; n=89.


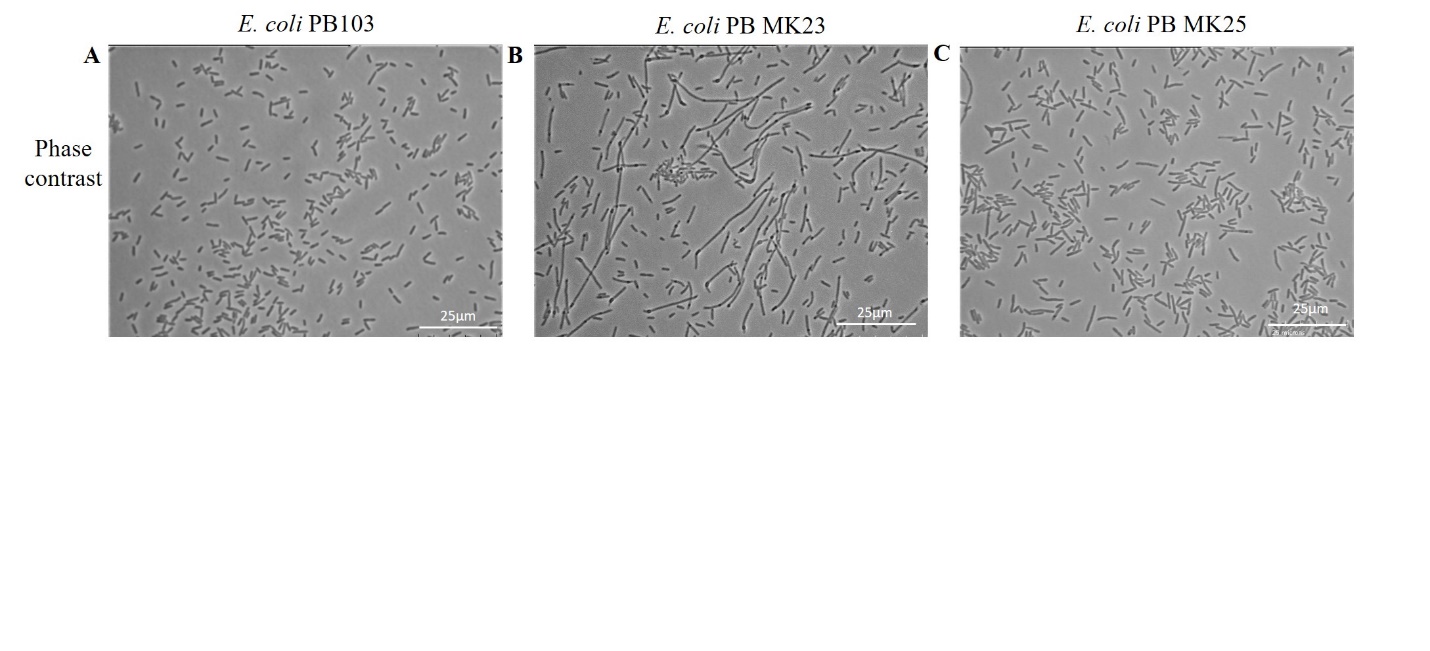


Figure S10. DivIVA_Ef_ exhibited loss of localization at the cell poles and midcell position in *E. faecalis* MWMR16 cells. Averaged images and fluorescence intensity traces of *E. faecalis* MWMR16 cells grown to mid-exponential phase in BHI broth and dual-stained with DAPI and Alexa-Fluor 488 as described in the methodology section and images were acquired using the InVitro 3 and ImagePro 6.0 softwares (Media Cybernetics) as described in Methodology. DivIVA_Ef_ with coiled-coil disrupted region localized along the cell membrane.


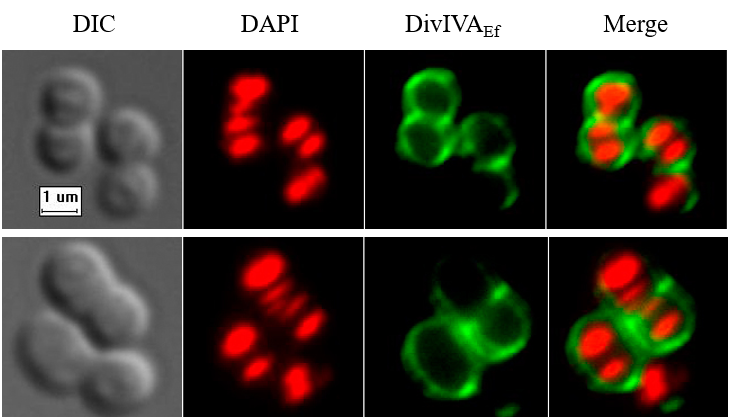

Supplement: Supplementary file 3 [file Data_Sheet_3.docx]
